# Niches for the Long-Term Maintenance of Tissue-Resident Memory T Cells

**DOI:** 10.3389/fimmu.2018.01214

**Published:** 2018-05-31

**Authors:** Shiki Takamura

**Affiliations:** Department of Immunology, Faculty of Medicine, Kindai University, Osaka, Japan

**Keywords:** distribution of memory T cells, maintenance of memory T cells, mucosal immunity, infectious immunity, vaccine

## Abstract

Tissue-resident memory T cells (T_RM_ cells) are a population of immune cells that reside in the lymphoid and non-lymphoid organs without recirculation through the blood. These important cells occupy and utilize unique anatomical and physiological niches that are distinct from those for other memory T cell populations, such as central memory T cells in the secondary lymphoid organs and effector memory T cells that circulate through the tissues. CD8^+^ T_RM_ cells typically localize in the epithelial layers of barrier tissues where they are optimally positioned to act as sentinels to trigger antigen-specific protection against reinfection. CD4^+^ T_RM_ cells typically localize below the epithelial layers, such as below the basement membrane, and cluster in lymphoid structures designed to optimize interactions with antigen-presenting cells upon reinfection. A key feature of T_RM_ populations is their ability to be maintained in barrier tissues for prolonged periods of time. For example, skin CD8^+^ T_RM_ cells displace epidermal niches originally occupied by γδ T cells, thereby enabling their stable persistence for years. It is also clear that the long-term maintenance of T_RM_ cells in different microenvironments is dependent on multiple tissue-specific survival cues, although the specific details are poorly understood. However, not all T_RM_ persist over the long term. Recently, we identified a new spatial niche for the maintenance of CD8^+^ T_RM_ cells in the lung, which is created at the site of tissue regeneration after injury [termed repair-associated memory depots (RAMD)]. The short-lived nature of RAMD potentially explains the short lifespans of CD8^+^ T_RM_ cells in this particular tissue. Clearly, a better understanding of the niche-dependent maintenance of T_RM_ cells will be important for the development of vaccines designed to promote barrier immunity. In this review, we discuss recent advances in our understanding of the properties and nature of tissue-specific niches that maintain T_RM_ cells in different tissues.

## Introduction

When naïve T cells encounter cognate antigen in the draining lymph node (LN), the cells are activated, initiate a proliferative program, and differentiate into a heterogeneous population of effector T cells. These effector T cells then home back to the site of infection and eliminate pathogen-infected cells. While most effector cells die after clearance of the pathogens, some cells subsequently differentiate into memory T cells. During the course of a T cell response, each T cell receives spatially and temporally distinct instructive signals that impact their ultimate fate; either death or differentiation into different types of memory cells with distinct functional and migratory properties (1, 2). For example, T cells primed by antigen-presenting cells (APC) with weak stimulatory potential preferentially remain in the LN and differentiate into central memory T cells (T_CM_ cells) where they survey lymph and blood (3, 4). On the other hand, T cells primed by APC with high stimulatory potential (e.g., strong costimulation) differentiate into potent effector cells that migrate to inflamed tissues and subsequently die (3). Effector cells that additionally receive tissue-specific instructive signaling differentiate into tissue-resident memory T cells (T_RM_ cells) and establish permanent residency within the tissues (1, 5). Effector T cells that fail to receive optimal tissue-instructive signals may differentiate into effector memory T cells (T_EM_ cells) that circulate between blood and certain peripheral tissues.

It is now appreciated that T_RM_ cells comprise the majority of memory T cells in the non-lymphoid tissues (NLT) and confer immediate protection against infection of barrier tissues (6). These cells are part of a comprehensive memory response that also include the T_CM_ and T_EM_ populations. T_CM_ cells exhibit high proliferative potential upon reactivation in the LN, thereby providing a major source of secondary effector cells that ultimately facilitate pathogen clearance (7). T_EM_ cells play a supportive role to T_RM_ by virtue of their immediate effector functions and their ability to rapidly traffic sites of infection (8). While the maintenance of circulatory memory T cell populations (T_CM_ and T_EM_) has been shown to depend on the homeostatic cytokines IL-7 and IL-15, the factors that regulate the maintenance of T_RM_ cells are ill defined. Furthermore, since T_RM_ cells in each tissue are maintained in distinct microenvironments, these cells must adapt to local cues for their long-term survival.

The external or internal surfaces of the body such as the skin and the mucosal linings of the gastrointestinal, respiratory, and urogenital tracts are a major gateway for infectious pathogens to access to the body. The surfaces of these barrier tissues are covered by different types of epithelial layers: from single layers of flattened or columnar cells to multiple layers of different types of epithelial cells. Each of these epithelial layers, along with the connective tissues that underlie the epithelium in each tissue, provide distinct microenvironments depending on their particular physiological and functional needs. The different types of immune cells that reside in these distinct microenvironments, such as macrophages, dendritic cells (DC), γδ T cells, and innate lymphoid cells (ILC), each adapt to these unique environments and play important roles in maintaining the integrity of these epithelial barriers (9–12). Accumulating evidence has revealed that the relationship between T_RM_ cells in these tissues and the original resident cell populations is dynamic and complex. For example, some tissue-resident immune cells interact with T_RM_ cells and provide niche factors for their maintenance (13–15). In other cases, tissue-resident immune cells and T_RM_ cells share local signals necessary for their long-term survival or compete with one another for access to niches that enable them to persist in the tissue (16). Furthermore, it is becoming clear that T_RM_ cells are also established in non-barrier tissues (such as the brain, liver, and kidney) as well as the primary lymphoid organs and secondary lymphoid organs (SLOs) and protect tissues from infectious pathogens disseminated by hematogenous or cellular (e.g., neural) pathways (17). The niches and factors that enable the maintenance of T_RM_ cells in these tissues differ significantly from those in the epithelial tissues. In this review, we discuss the distribution of T_RM_ cells in each tissue and the factors that influence the establishment and maintenance of T_RM_ cells.

## Non-Lymphoid Organs

### Barrier Tissues

#### Skin

The skin is comprised of three main layers: the epidermis, dermis, and subcutaneous fatty region. The epidermis and dermis are separated by a basement membrane and harbor numerous unique populations of innate and adaptive immune cells. Many of these cells are resident populations and form a sophisticated immune network that provides a biological barrier against invading pathogens (18).

The epidermis is an avascular tissue composed primarily of keratinocytes (19). Dead keratinocytes comprise the outmost layer of the epidermis, known as the stratum corneum, and serve as a physiological barrier (20). Keratinocytes in the deeper layers, such as the stratum granulosum and stratum spinosum, provide integrity to the skin and play multiple roles in the initiation of local immunity by recognizing pathogens through pattern recognition receptors and by secreting a wide variety of cytokines and chemokines (21). These cells also secrete various factors necessary for the development and homeostasis of immune cells residing in the epidermis (21). The bottom layer, the stratum basale, consists primarily of a single layer of basal cells—precursors of the keratinocytes that comprise the upper layers of the skin (22). The hair follicles also consist of keratinocytes and provide unique niches for immune cells including T_RM_ cells (23).

At least three immune cell types are maintained in the epidermis: Langerhans cells (LC), dendritic epidermal T cells (DETC) expressing γδ T cell receptors (TCR), and memory T cells expressing αβ TCR. These cells do not recirculate under steady-state conditions, exhibit a dendritic morphology, and inhabit several anatomical as well as physiological niches for their development and maintenance (20).

Langerhans cells are present in all layers of the epidermis, especially in the stratum spinosum, and are the only APC in the epidermis under steady-state conditions (24). The development and maturation of LC depends on transforming growth factor-β (TGF-β), which is secreted by keratinocytes, DETC (paracrine), and the LC themselves (autocrine) (24). Although TGF-β1 is secreted as a latent (inactive) form, it is trans-activated by integrin α_v_β_6_ and α_v_β_8_ expressed on keratinocytes in the interfollicular regions and near the hair follicles (25, 26). TGF-β has also been shown to be required for the retention of LC within the epidermis since the loss of TGF-β1 signaling leads to the spontaneous migration of LC to the regional LN (25). In addition to initiating adaptive immune responses, LC are also involved in the induction of tolerance by promoting the proliferation of regulatory T (Treg) cells in the epidermis under steady-state conditions (27).

In mice, DETC comprise a large proportion of immune cells in the epidermis (20). DETC are distributed throughout the epidermis, secrete a variety of cytokines, chemokines, and growth factors, and play key roles in the wound repair, tumor surveillance, and inflammation (28). They persist in the epidermis for life and are maintained by homeostatic turnover. Common γ-chain signaling through IL-7 and IL-15, as well as signaling *via* the aryl hydrocarbon receptor (AhR) are known to be required for the development and maintenance of DETC (29–32). This is consistent with the fact that AhR ligands are abundant in the skin since they are formed from tryptophan *via* ultraviolet radiation (33). In contrast to LC, the maintenance of DETC is independent of TGF-β (34).

**Figure 1:**
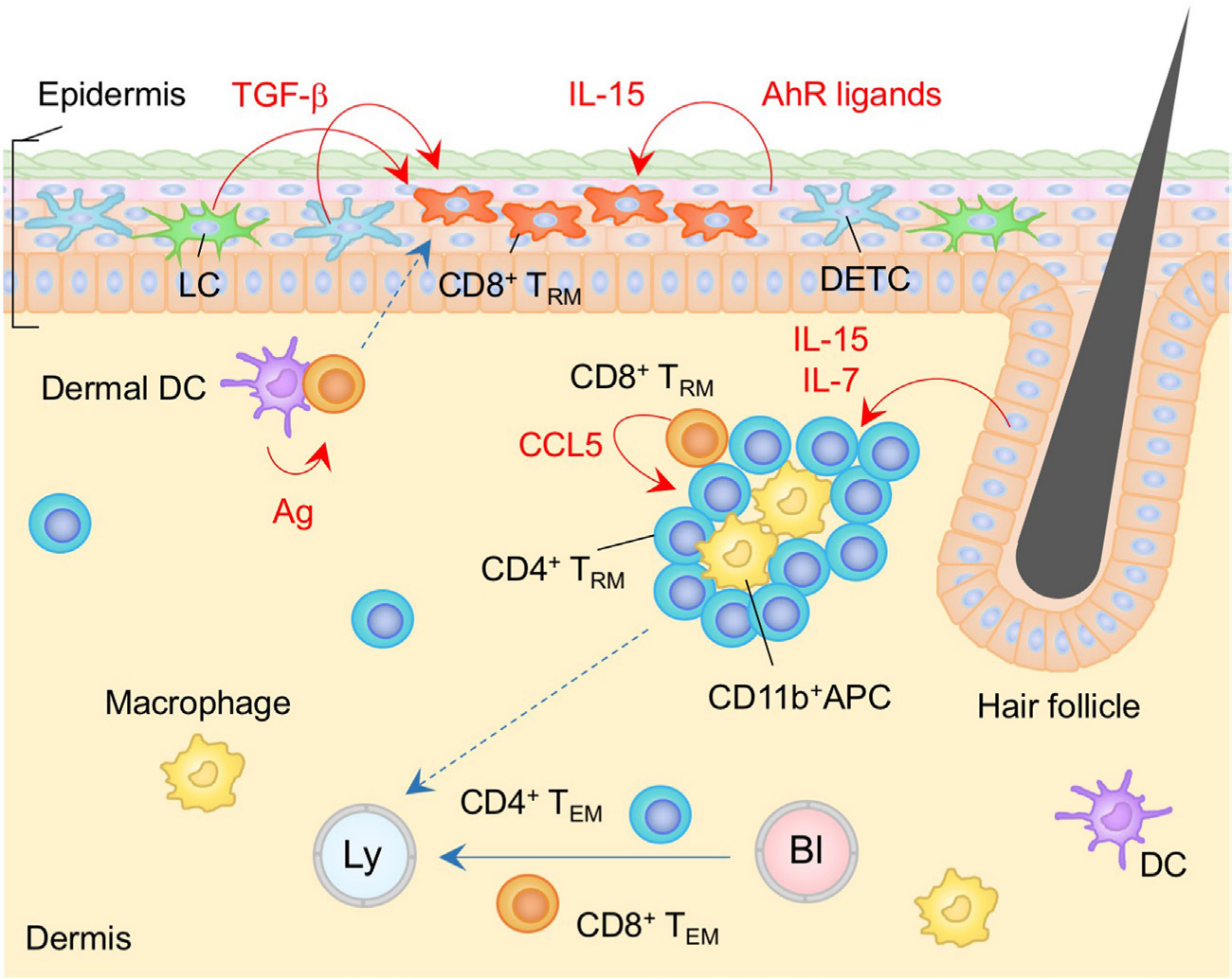
T_RM_ niches in the skin. Langerhans cells (LC), dendritic epidermal T cells (DETC) expressing γδ T cell receptors, and CD8^+^ T_RM_ cells are maintained in the epidermis. CD8^+^ T_RM_ cells displace epidermal niches originally occupied by DETC at the site of infection. Transforming growth factor (TGF)-β secreted from LC and DETC, IL-15, and aryl hydrocarbon receptor (AhR) ligands play a role in the generation and maintenance of epidermal CD8^+^ T_RM_ cells. Memory CD4^+^ T cells in the dermis form clusters with CD11b^+^ APC around the hair follicles. CCL5 secreted from peri-collicular CD8^+^ T cells promotes formation of clusters. Although most memory CD4^+^ T cells in the cluster exhibit canonical T_RM_ phenotypes, long-period parabiosis experiments revealed that this population is slowly replenished by cells from the circulation. IL-7 and IL-15 secreted from keratinocytes in the hair follicles promote T cell persistence in the cluster. T_EM_ cells are passing through the dermis. Orange and blue cells indicate CD8^+^ and CD4^+^ T_RM_ cells, respectively, unless otherwise stated. Red lines indicate the representative niche factors that influence the maintenance of T_RM_ cells. Blue lines indicate the migratory routes. Dashed lines indicate potential impact of niche factors (red) or migratory routes (blue). Abbreviations: Ly, lymph vessel; Bl, blood vessel; Ag, antigen; APC, antigen-presenting cell; T_RM_, tissue-resident memory T cells; T_EM_, effector memory T cells.

The majority of αβ T cells that reside in the epidermis are CD8^+^ T_RM_ cells (35) (Figure 1). These cells express canonical T_RM_ makers such as the activation marker CD69, the E-cadherin-binding integrin CD103, and the collagen-binding integrin CD49a, in the absence of cognate antigen signaling (36, 37). Although CD8^+^ T_RM_ cells are widely found throughout the body (38), their numbers are generally elevated at sites of infection and/or inflammation (37, 39, 40). Several chemokines are known to be involved in the recruitment of CD8^+^ T_RM_ precursors (KLRG1^lo^) into the epidermis, including cutaneous T cell-attracting chemokine (CTACK), CXCL9 and CXCL10. CTACK is constitutively expressed by epidermal keratinocytes and attracts CCR10 expressing T cells (41). Since memory T cells do not express CCR10, it is likely that CTACK primarily drives the recruitment of effector T cells to the epidermis, but not the retention of memory T cells at that site (42). Other inflammatory chemokines, such as CXCL9 and CXCL10, are highly expressed by keratinocytes in response to infection, and facilitate the recruitment of CXCR3^+^ memory precursor effector CD8^+^ T cells to the epidermis (43). Like LC, these cells subsequently receive TGF-β signals upon arrival, which is a critical factor for the upregulation of the E-cadherin binding integrin, CD103 (43) (Figure 1). Since E-cadherin is expressed on epithelial cells, including keratinocytes, it is likely that the upregulation of CD103 facilitates the retention of T cells in the epidermis (44). TGF-β signaling also downregulates the T-box family protein T-bet and eomesodermin, a process of which facilitates T_RM_ cell development (45). CCR8 expression is also upregulated following the migration of T cells into the epidermis by yet unidentified factors derived from keratinocytes. It appears likely that this chemokine receptor also facilitates the maintenance of cells within the epidermis (46, 47). Finally, there may also be a role for CXCR6 in the maintenance of T_RM_ in the epidermis since its absence results in a marked reduction in the number of skin CD8^+^ T_RM_ (42).

CD8^+^ T_RM_ cells in the epidermis display a unique dendritic morphology (16, 35, 48), which is distinct from that of LC and DETC (20, 48). Epidermal CD8^+^ T_RM_ cells are located in the basal layers of the epidermis and slowly but continuously migrate between keratinocytes, while LC and DETC are mostly immotile (16, 48). Importantly, Zaid et al. have demonstrated a substantial decrease in the numbers of DETC and a concomitant increase in the numbers of CD8^+^ T_RM_ cells at the site of infection, indicating the strict competition between DETC and CD8^+^ T_RM_ cells for the epidermal niches (16) (Figure 1). Furthermore, both of these populations also depend on locally produced homeostatic signals, such as IL-15 and AhR ligands, for their long-term maintenance (16, 30, 32, 43). These common features may explain the stable persistence of CD8^+^ T_RM_ cells within the epidermal niches for many years without repopulation by DETC (16). Furthermore, the relatively higher numbers of αβ T cells, as compared to γδ T cells, in the human epidermis might be a consequence of the persistent occupation of epidermal niches by CD8^+^ T_RM_ cells generated by prior infection and/or inflammation (20). It is important to note here that the capacity of epidermal T_RM_ niches are extremely large (approximately 7 × 10^3^ T cells/cm^2^) (49). The high capacity of epidermal niches allows the *de novo* establishment of T_RM_ cells with different specificities without displacement of pre-existing T_RM_ cells after rechallenge. Importantly, this allows T_RM_ cells with multiple specificities to be stably maintained in the epidermis (49). By contrast, γδ T cells are displaced by CD8^+^ T_RM_ cells even when the number of T_RM_ cells relatively low, suggesting an occupational advantage for CD8^+^ T_RM_ cells over γδ T cells in the epidermal niches. Finally, since the environment in which epidermal CD8^+^ T_RM_ cells persist has limited access to blood-derived signals as well as nutrients, these cells uniquely express fatty acid transporters, Fabp4 and Fabp5, and rely on extracellular fatty acid for their survival (50).

The dermis that underlies the basement membrane is composed mainly of fibroblasts and the extracellular matrix (a network of collagen and elastin fibers). Heterogeneous populations of immune cells, including αβ T cells, γδ T cells, subsets of DC, macrophages, mast cells, and ILC are all found in the dermis (21). The dermis also contains both lymphatic and blood vessels, providing a source of T_EM_ cells that are transiting through the tissues.

In contrast to the situation in the epidermis, most αβ T cells located in the dermis are CD4^+^ T cells, including both conventional T cells and Treg (14, 35, 51, 52). These cells display an amoeboid morphology and traffic rapidly through the dermis (35). Long-period parabiosis experiments (12–16 weeks) using naïve animals has revealed that a large fraction of CD4^+^ T cells recruited from the circulation acquire the expression of CD69 and CD103 following entry into the skin (14). Of note, T_RM_-phenotype CD4^+^ T cells in the dermis are tissue-circulating T_EM_ cells despite their relatively slow turnover rate, as the ratio of host and partner CD4^+^ T cells was equilibrated in these parabiosis experiments (14). These CD4^+^ T cells form clusters with CD11b^+^ APC around hair follicles (14) (Figure 1). The numbers of hair follicle-associated clusters, as well as the numbers of CD4^+^ T cells within each cluster, are increased following local infection and/or inflammation, indicating that tissue conditioning creates new dermal CD4^+^ T cell niches (14). CCL5 secreted from peri-follicular CD8^+^ T cells promotes the formation of the CD4^+^ T cell clusters (14). In addition, IL-7 and IL-15 are predominantly secreted by unique population of keratinocytes in the hair follicles, helping to sustain T cell persistence within the cluster (23). Such unique structures are potentially identical to the classical inducible skin-associated lymphoid tissues that provide both spatial and physiological niches for the maintenance of memory T cells (53).

Although local tissue instructions promote the formation of T_RM_ in the absence of local antigen (37), recent studies have revealed that encounters with cognate antigen at the site of infection significantly enhance the establishment of CD8^+^ T_RM_ cells in the skin, presumably in the epidermis (54). While several cell-intrinsic mechanisms of T_RM_ formation induced by an antigen-driven “second hit” are suggested (5), one certain outcome is the upregulation of CD69 (54). It has been established that T cells recruited to peripheral tissues upregulate sphingoshin-1-phosphate receptor 1 (S1P_1_), and sense the gradient of sphingoshin-1-phosphate (S1P) (55), which guides T cells to the draining lymphatics of the tissue. Surface expression of CD69 antagonizes the expression of S1P_1_ (56), thereby inhibiting the egress of T cells from the skin (57). Since lymphatic vessels are not found in the epidermis, it is likely that the second antigen hit and the resultant retention induced by CD69-mediated inhibition of S1P_1_ occurs in the dermis, and subsequently promotes the establishment of CD8^+^ T_RM_ in the epidermis. In support of this concept, APC in the skin function as a gatekeeper for the development of CD8^+^ T_RM_ cells, such that CD8^+^ T cells with distinct antigen specificities compete for APC as a source of second hit signaling, leading to the selection of dominant epitope-specific CD8^+^ T cells (58). This leads to the reduced formation of CD8^+^ T_RM_ cells specific for subdominant epitopes since these T cells presumably fail to receive second antigen hit signaling and rapidly egress from the dermis. Such antigenic selection may be the underlying mechanism driving the accumulation of highly functional, melanocyte antigen-specific CD8^+^ T_RM_ cells in the vitiligo-affected skin (59, 60). It is important to note that transcriptional downregulation of *Klf2*, as well as its downstream target *S1pr1* (which encodes S1P_1_), is also induced by several cytokines such as TGF-β, IL-33, and tumor necrosis factor (TNF), even in the absence of local antigen (61). However, certain factors that enable the acquisition of a unique transcription profile defining T_RM_ cells, including the upregulation of *Hobit* and *Blimp1*, have not been not fully elucidated (62, 63).

#### Gut, Intestine

The intestinal mucosa consists of a single layer of intestinal epithelial cells that overlies the lamina propria (LP), a thin layer of loose connective tissue. The epithelium and LP are separated by a basement membrane and each provides a distinct immunological niche for the maintenance of T_RM_ cells.

The diverse populations of immune cells embedded within the intestinal epithelium are referred to as intestinal intraepithelial lymphocytes (IELs). The greatest concentration of IEL is located in the small intestine (SI) where there are approximately 10–15 IEL per 100 epithelial cells. This ratio of IEL to epithelial cells gradually decreases along the intestines, such that the colon hosts relatively few IEL (64). The differences in the relative numbers of IEL in each intestinal compartment likely reflects regional differences in the anatomy of the villi, the intestinal microenvironment (including microbiota), and the composition of epithelial cells (e.g., enterocytes, goblet cells, Paneth cells, enteroendocrine cells, and stem cells). Epithelial cells are a dynamic population and cells situated at the top of the villi typically die within 3–5 days and are continually replaced by new cells generated from the progenitor cells located in the crypt. Despite the short lifespan of epithelial cells, IEL are resident and do not recirculate (65).

Intraepithelial lymphocytes in the intestines are primarily T cells, although there is also a small population of cells that are negative for TCR, such as ILC-like cells (66). IEL T cells can be divided into two subsets, referred to as peripheral and thymic. Peripheral IEL (type a, induced or conventional) are derived from antigen-experienced CD8^+^ or CD4^+^ T cells that have homed to the epithelium. Thymic IEL (type b, natural or unconventional) express CD8α homodimers with either TCRαβ or TCRγδ, and migrate from the thymus to the epithelium shortly after birth (67). In mice, thymic IEL dominate in the SI while peripheral IEL dominate in the colon (64). The overall ratio of thymic to peripheral IEL declines with age, although the total number of IEL remains relatively constant (67, 68), suggesting that the two types of IEL share the same spatial niche in the epithelium. However, there is a severe reduction in the numbers of peripheral but not thymic IEL in germ-free animals (69), suggesting that the physiological niches that maintain peripheral and thymic IEL must differ in some way. This review will focus on peripheral IEL.

**Figure 2:**
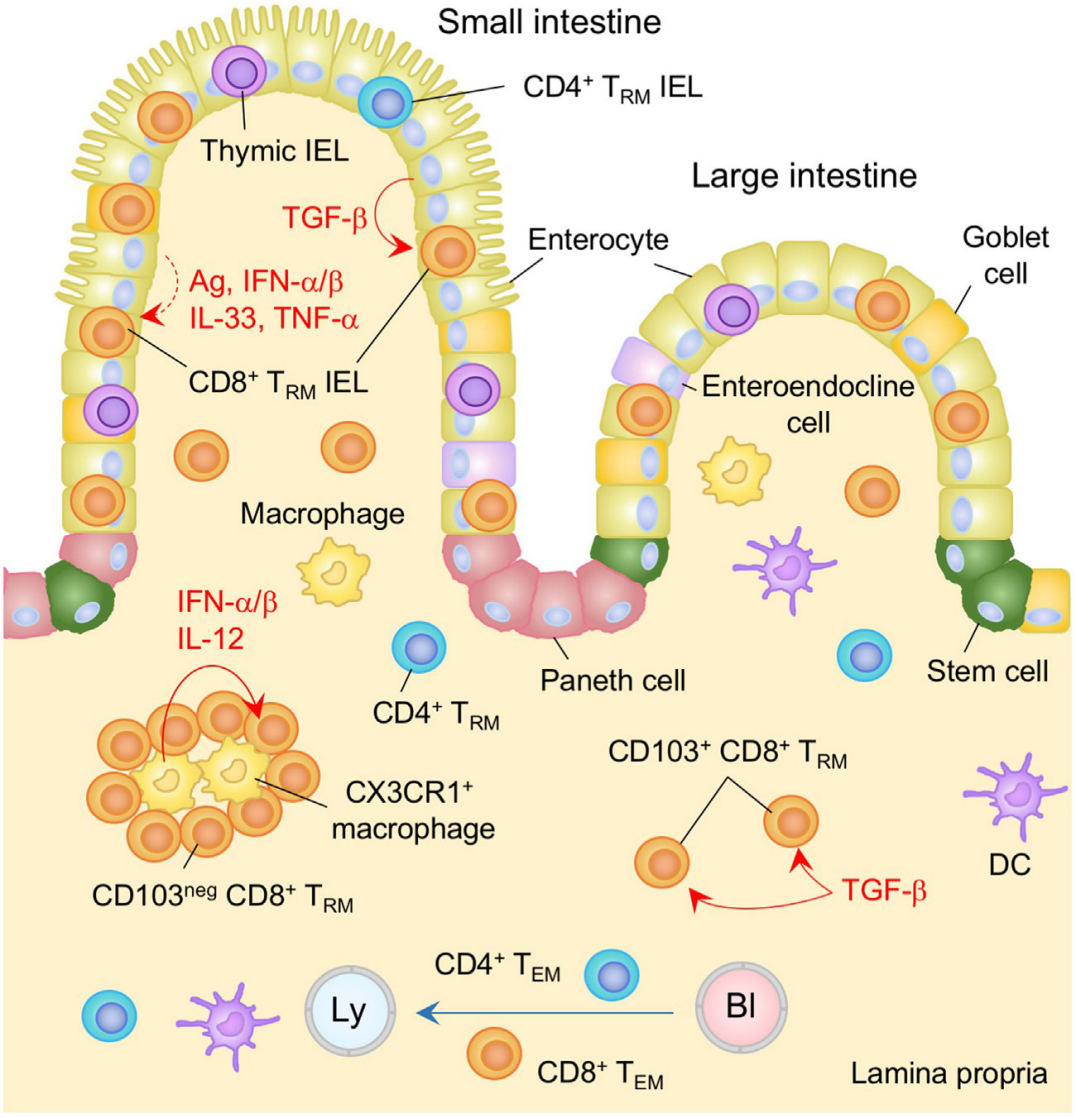
T_RM_ niches in the in the intestine. Large numbers of CD8^+^ T_RM_ cells and few numbers of CD4^+^ T_RM_ cells are present in the intestinal intraepithelial lymphocyte (IEL) compartment. TGF-β is constitutively available in the intestinal epithelium and promotes the generation of T_RM_ cells in this compartment by upregulating CD103 as well as Runx3. Either cognate antigen or inflammatory cytokines is required for upregulation of CD69 on epithelial T_RM_ cells. Both TGF-β-dependent (CD103^+^) and independent (CD103^−^) populations of CD8^+^ T_RM_ cells present in the lamina propria (LP). The latter form cluster with CX3CR1^+^ macrophages. Interferon (IFN)-α/β and IL-12 secreted by macrophages control the size of the cluster. T_EM_ cells are passing through the LP. Orange and blue cells indicate CD8^+^ and CD4^+^ T_RM_ cells, respectively, unless otherwise stated. Red lines indicate the representative niche factors that influence the maintenance of T_RM_ cells. A blue line indicates the migratory route. A dashed line indicates potential impact of niche factors. Abbreviations: Ly, lymph vessel; Bl, blood vessel; Ag, antigen; neg, negative; T_RM_, tissue-resident memory T cells; T_EM_, effector memory T cells.

Significant numbers of antigen-specific T_RM_ cells are established in the intraepithelial compartment following intestinal infections (70–72) (Figure 2). The majority of these cells are CD8^+^ T cells, although smaller numbers of CD4^+^ T cells are also observed (73). Interestingly, a large number of memory-like γδ T cells is also generated following intestinal infection. However, these cells are rarely found in the IEL compartment, suggesting that CD8^+^ T_RM_ cells but not γδ T cells are preferentially lodged in the intraepithelial niches (74). Nearly all CD8^+^ T_RM_ cells in this compartment express CD69 and CD103 (70, 71) and are scattered within the epithelium. Recruitment of effector cells to this site, including T_RM_ precursors, is governed by the α4β7 and CCR9 integrins, both of which are upregulated on T cells, mainly in response to retinoic acid (a vitamin A metabolite) which is present during priming in the intestinal inductive sites (75). The α4β7 integrin facilitates the extravasation of the cells from the venules in the LP (76, 77). CCR9 is required for T cell migration to the SI (78, 79), since its ligand, CCL25, is constitutively expressed by epithelial cells in the SI but not the colon (80).

As with thymic IEL, a process of tissue adaptation takes place following recruitment of peripheral CD8^+^ T cells into the epithelium. Specifically, the local environment promotes the differentiation of effector T cells into T_RM_ and facilitates their subsequent retention at that site. In this regard, TGF-β, which is constitutively available at the intestinal epithelium (81, 82) (Figure 2), induces the upregulation of CD103 on recent immigrants. Consistent with this, the lack of CD103 or the TGF-β receptor on T cells is correlated with a significant defect in the accumulation of both peripheral and thymic IEL within the intestinal epithelium (71, 83–85). By contrast, overexpression of TGF-β results in increased proportion of thymic IEL in the SI (86), highlighting the non-redundant, regulatory role of TGF-β in the number of T_RM_ cells retained in the intestinal epithelium. TGF-β signaling also induces the expression of Runx3 (87–89), which has been identified as a master regulator of tissue residency (90). Although the precise role of Runx3 in retaining cells in the SI is not yet clear, it is known to promote the expression of CD8αα (88), which binds to the thymus leukemia antigen that is constitutively expressed on the intestinal epithelium (91). Interestingly, TGF-β-independent populations of T_RM_ cells also accumulate in the IEL compartment during chronic infection with lymphocytic choriomeningitis virus (LCMV) (85). These cells do not express CD103 and are thought to represent recent arrivals that are recruited continually from the circulation upon activation with persistent viral antigens (85).

While CD8^+^ T_RM_ IEL are associated with gut infection, they are also established following systemic infections (6, 83, 85, 90, 92, 93), and their numbers are especially robust under lymphopenic conditions (e.g., Rag^−/−^) (83, 93, 94). IEL generated through systemic immune responses exhibit canonical T_RM_ phenotypes (CD69^+^ CD103^+^) despite the absence of TCR signaling (as determined by the lack of Nur77 expression) (83), indicating that cognate antigen is not required for the upregulation of CD69 in the gut. In fact, some cytokines that can be secreted in the epithelium, such as IL-33, interferon-α/β (IFN-α/β), and TNF-α, are known to contribute to the antigen-independent upregulation of CD69 (83). Nevertheless, the number of CD8^+^ T_RM_ cells established in the intestinal epithelium following systemic priming is significantly less than that generated by gut infection (71). This is largely due to the relatively poor accumulation of memory precursor cells into the intestinal epithelium following non-intestinal infection (71). While significant progress has been made in understanding gut T cell memory, the impact of infection-driven tissue conditioning on the spatial as well as the physiological niches (local antigen and cytokine milieu) on the maintenance of T_RM_ cells in the intestinal epithelium is largely unknown.

The homeostatic cytokine IL-15 is constitutively produced by intestinal epithelial cells in response to signaling through MyD88, suggesting that there is a background level of stimulation by intestinal microflora (95). As with DETC in the skin epidermis, the development and maintenance of thymic IEL depends on local signaling *via* IL-15, as lack of this signaling results in the loss of more than 90% of thymic IEL (96–98). Although it has been proposed that IL-15 produced by inflamed mucosal tissues accelerates the accumulation of circulating effector CD8^+^ T cells in the SI through the upregulation of the mammalian target of rapamycin and T-bet (93), survival of CD8^+^ T_RM_ cells in most peripheral tissues, including the SI (both in the epithelial compartment and LP), is independent of IL-15 (99). This suggests that the physiological niches inhabited by peripheral and thymic IEL exhibit different characteristics.

The LP harbors the vast majority of immune cells in the body. These cells are located in organized lymphoid structures, termed gut-associated lymphoid tissues, such as Peyer’s patches (PP), cecal patches, colonic patches, cryptopatches, and solitary isolated lymphoid tissues (100). Large numbers of T cells are present throughout the LP. T-cell homing to small intestinal LP is mediated by integrin α4β7 and CCR9, whereas the orphan G-protein-coupled receptor 15 is required for migration of T cells to the large intestinal LP (101). Once in the relevant gut site, T cells receive instructive signals for their full differentiation into T_RM_ cells. Note that a stable population of memory-like γδ T cells is established in the LP, suggesting limited competition of anatomical niches between T_RM_ cells and γδ T cells in this compartment (74).

In contrast to memory T cells in the IEL compartment, memory cells located in the LP include both T_EM_ and T_RM_ (Figure 2). This is because the LP contains both lymphatic drainage and blood supplies (65) and suggests that T_RM_ cells in this tissue need to continually resist tissue egress signals for their long-term maintenance. CD69 is expressed on a large proportion of T cells in the LP (13, 65, 70, 71, 73, 83, 85), and plays a key role in antagonizing S1P_1_-mediated tissue egress. As with the IEL compartment, T cells in the LP express CD69 despite the absence of cognate antigen (83). In support of this, parabiosis experiments have revealed that although partner-derived cells include sizable proportion of CD69^−^ cells (which represent transients in the LP), nearly 80% of CD8^+^ T cells recruited from the partner become CD69^+^ following arrival (65), indicating the influence of constitutively secreted inflammatory cytokines in this tissue (83). However, the ratios of host and partner CD8^+^ T cells in the LP as well as the epithelium never become fully equilibrated following parabiosis, indicating the limitation of local instructive signaling for the formation of T_RM_ cells in those tissues under steady-state conditions (65).

Following recruitment to the LP, T cells downregulate integrin α4β7, indicating that integrin α4β7 is not required for their retention (83). Instead, a proportion of CD8^+^ T cells upregulate CD103 in a TGF-β-dependent manner (70, 71, 83, 85). These cells form a resident population and are scattered throughout the LP (70) (Figure 2, shown as CD103^+^ CD8^+^ T_RM_). Interestingly, CD103^−^ cells are also found to be resident in the LP (these cells are refractory to depletion by a systemically introduced antibody) (70), suggesting the presence of CD103-independent retention signals. These cells form clusters with CX3CR1^+^ macrophages primarily located under the crypts and the size of this population is independent of TGF-β, but is controlled by type I IFN and IL-12 (13) (Figure 2). Since these cytokines are provided mainly by monocyte-derived CCR2^+^ macrophages that have been recruited in response to local infection, and *Cxcr3*-deficient CD8^+^ T cells fail to form clusters (13), it is reasonable to conclude that infection-induced tissue conditioning facilitates the development of CD103^−^ CD8^+^ T_RM_ population. However, the accumulation of CD103^−^ CD8^+^ T_RM_ cells is also evident even in the absence of intestinal infection (83, 85), suggesting the presence of additional niches that sustain CD103^−^ CD8^+^ T_RM_ cells in the infection/inflammation-inexperienced LP.

#### Female Reproductive Tract (FRT)

**Figure 3:**
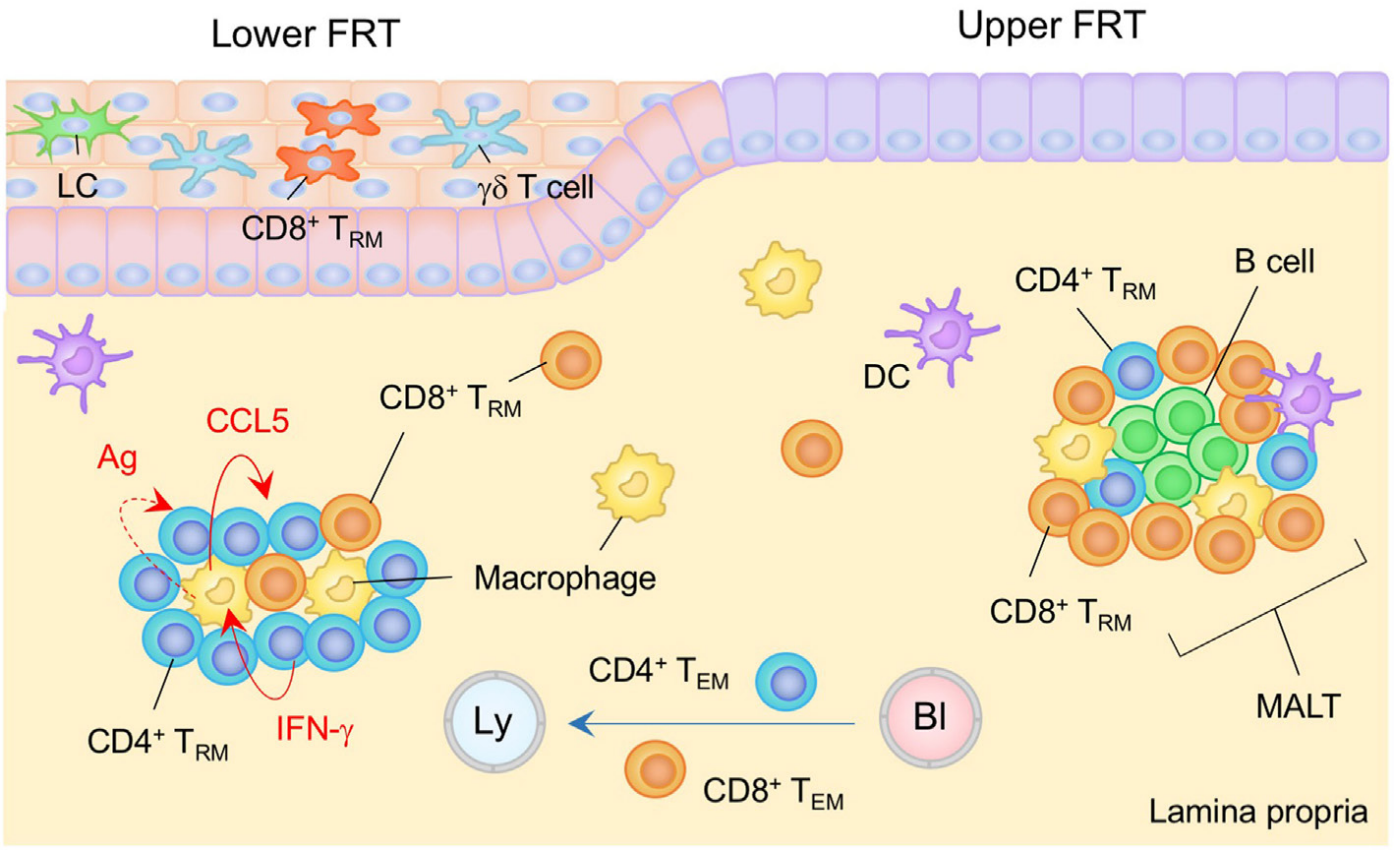
T_RM_ niches in the in the female reproductive tract (FRT). The FRT consist of the upper (endometrium and endocervix) and lower (vagina and ectocervix) reproductive tract. The upper FRT is composed of type I epithelia while the lower FRT is composed of type II epithelia. Mucosa-associated lymphoid tissues (MALT) are found in the lamina propria (LP) of the upper FRT. Both CD4^+^ and CD8^+^ T_RM_ cells are involved in the MALT. The size of the MALT is regulated by the phase of the menstrual cycle. In the lower FRT, CD8^+^ T_RM_ cells present mainly within the epithelial layers. Antigen is not required for the establishment of CD8^+^ T_RM_ cells in this tissue. CD4^+^ T_RM_ cells usually form clusters with macrophages in the LP. The structure of the cluster is sustained by a constitutively produced cytokine/chemokine network in which IFN-γ secreted by CD4^+^ T_RM_ cells drives CCR5 production by macrophages, which attracts and retains CD4^+^ T_RM_ cells within the cluster. Cognate antigen may be involved in driving CD4^+^ T cell production of IFN-γ. T_EM_ pass through the LP. Orange and blue cells indicate CD8^+^ and CD4^+^ T_RM_ cells, respectively, unless otherwise stated. Red lines indicate the representative niche factors that influence the maintenance of T_RM_ cells. A blue line indicates the migratory route. A dashed line indicates potential impact of niche factor. Abbreviations: Ly, lymph vessel; Bl, blood vessel; Ag, antigen; LC, Langerhans cells; T_RM_, tissue-resident memory T cells; T_EM_, effector memory T cells.

The mucosal surfaces of FRT can be divided into two types, referred to as type I and type II. The upper FRT, such as endometrium and endocervix, expresses type I mucosal surfaces, which are covered by a single layer of columnar epithelial cells linked by tight junctions. The lower FRT, such as the vagina and ectocervix, expresses type II mucosal surfaces, which are covered by multiple layers of non-keratinized stratified squamous epithelium binding to a basement membrane (102). Mucosa-associated lymphoid tissues (MALT) are found in the stromal layer (lamina propria) and the submucosa of the upper but not the lower FRT (103) (Figure 3). Migration of effector, as well as memory, T cells into the mucosa of the FRT is significantly restricted in the absence of local infection and/or inflammation (104). Once recruited, however, T_RM_ cells are formed and maintained in both compartments under the control of local environmental cues.

The endometrium is a highly dynamic tissue in women. It undergoes remarkable cyclical changes of growth, differentiation, and degeneration under the control of the hormones estrogen and progesterone. The spontaneous decidualisation of the endometrial epithelium and stroma, which causes menstruation, and subsequent re-epithelization of endometrium periodically occurs (105), suggesting that limited, if any, anatomical niches are available for the long-term maintenance of T_RM_ cells. Yet, numerous immune cells, including memory T cells, are found along the stroma/submucosa of the upper FRT (106, 107). During the proliferative phase of the menstrual cycle, uterine immune cells become condensed, leading to a formation of lymphoid aggregates (107). These lymphoid aggregates, which are presumably identical to the MALT described above, mainly consist of a B cell core surrounded by memory CD8^+^ T cells and macrophages (107, 108) (Figure 3). The size of the MALT varies with the phase of the menstrual cycle, rising to 3,000–4,000 cells during the secretory phase and declining to 300–400 cells during the proliferative phase (109). This implies that there must be endocrine regulation of the T_RM_ niches. It is also known that CD8^+^ cytotoxic T lymphocyte (CTL) activity is suppressed during the secretory stage, presumably to minimize the recognition and rejection of allogenic sperm and the semi-allogenic fetus (107). Thus, the deployment of memory CD8^+^ T cells within the MALT in the uterine stroma/submucosa but not epithelial layer is organized to maintain reproductive function.

Recently, intravital imaging of the perimetrium and myometrium of the fallopian tubes has demonstrated the establishment of antigen-specific CD8^+^ T_RM_ cells in the upper FRT following resolution of virus infection at the uterus (110). The velocity of CD8^+^ T_RM_ cells in the uterine stroma (~10 μm min^−1^) is similar to that of CD8^+^ T_CM_ cells in the LN and is significantly higher than that of CD8^+^ T_RM_ in the skin epidermis (~2 μm min^−1^) (35, 110, 111). Since uterine CD8^+^ T_RM_ cells display poor dendritic morphology, as compared to skin CD8^+^ T_RM_ cells, and are found in a site where immune cells are present at relatively high density (35, 110, 112), it is likely that the CD8^+^ T_RM_ cell niches in the upper FRT exist within the MALT in the uterine stroma/submucosa. Furthermore, an experimental *Chlamydia* vaccine that promotes antigen presentation by immunogenic CD11b^+^ CD103^−^, but not tolerogenic CD11b^−^ CD103^+^ DC, elicits stable CD4^+^ T_RM_ cell populations in the upper FRT. These cells provide significant protection against subsequent *Chlamydia* infection (113). The integrins α4β1 and α4β7 are involved in the migration of effector CD4^+^ T cells to this site as blockade of integrin α4 blocks uterine T cell homing during the early phase of infection (113–115). Large numbers of CD4^+^ T cells are recruited to the uterine stroma/submucosa after local infection with *Chlamydia* (116) and form clusters that also include small numbers of B cells and CD8^+^ T cells (117, 118). This indicates that CD4^+^ T_RM_ cells in the upper FRT are also maintained in MALT structures (Figure 3). B cells in the cluster also act as APC to CD4^+^ T cells, leading to the selection and maintenance of highly protective CD4^+^ T_RM_ cells (108, 119).

The immune cell composition of the lower FRT (type II epithelia) is basically similar to that of the skin: LC and γδ T cells survey the epithelium, while heterogeneous subsets of DC and macrophages survey the LP (103). Although the lower FRT does not contain MALT in the steady state, both CD8^+^ and CD4^+^ T_RM_ cells can be established in the lower FRT following intravaginal infections, such as those mediated by herpes simplex virus type 2 (HSV-2). Notably, after the clearance of the infection, memory CD4^+^ T cells, B cells, DC, and macrophages form clusters beneath the epithelial layer of the vagina (120) (Figure 3). CD4^+^ T_RM_ cells are predominantly distributed within the clusters, and their structures are sustained by a constitutively produced cytokine/chemokine network. IFN-γ secreted by CD4^+^ T_RM_ cells drive CCL5 production by macrophages which attracts and retains CD4^+^ T_RM_ cells within the cluster (15). Residual antigen may be involved in driving CD4^+^ T_RM_ cell production of IFN-γ (15). Although CD4^+^ T_RM_ cells are crucial for full protection against HSV-2 infection (15), establishment of CD4^+^ T_RM_ cells in the vaginal mucosa increases susceptibility to subsequent human immunodeficiency virus infection due an increase in the number of susceptible target cells (121, 122).

As with the skin epidermis, antigen-specific CD8^+^ T_RM_ cells reside within the epithelium and LP of the vaginal mucosa (123–125). T_RM_ cells in the vaginal LP are predominantly found in clusters (15). Migration of effector CD8^+^ T cells to the vaginal epithelium largely depends on CXCR3, a receptor for inflammatory chemokines CXCL9 and CXCL10 (126). IFN-γ secreted by arriving CD4^+^ T cells triggers production of those chemokines at the site of infection, demonstrating the importance of CD4^+^ T cells in promoting anti-viral CD8^+^ T cell responses in the FRT (126). Topical administration of these chemokines can effectively recruit circulating effector, but not memory, CD8^+^ T cells primed at a remote site to the genital mucosa even in the absence of cognate antigen, a strategy known as “prime and pull.” This leads to the establishment of long-term populations of CD8^+^ T_RM_ cells in the vagina (127). Interestingly, although effector CD4^+^ T cells are also recruited to the genital mucosa following prime and pull strategies, memory CD4^+^ T cells are not retained for the long term within the vagina (127), implying that the maintenance of CD4^+^ T_RM_ niches (the clusters in the vaginal LP) relies on local antigen. By contrast, and similar to the skin CD8^+^ T_RM_ cells that populate epidermal niches for DETC (16), CD8^+^ T cells recruited to the vaginal mucosa may occupy unique niches that were originally occupied by other resident cell types, such as γδ T cells in the epidermal layer of the vagina. Distinct from the skin CD8^+^ T_RM_ cells, however, the development and maintenance of CD8^+^ T_RM_ cells in the FRT is IL-15-independent (99). Currently, the factors that regulate the maintenance of T_RM_ cells in the FRT are largely unknown.

#### Upper Respiratory Tract (URT) and Lower Respiratory Tract (LRT)

The respiratory tract is divided into two compartments; the URT, comprised of the nasal cavities, pharynx, and larynx, and the LRT, comprised of the trachea, primary bronchi, and lungs. Although most studies have largely focused on T_RM_ cells in the LRT, most common airborne pathogens in the human primarily infect the URT. Thus, understanding the T_RM_ niches in both compartments is key for the development of vaccines that confer protection against respiratory pathogens.

The mucosal surface of the URT is comprised of pseudostratified ciliated columnar epithelial cells and an underlying LP. In mice, nasal-associated lymphoid tissues (NALT), the murine equivalent of the tonsils in human, are embedded directly in the submucosa at the base of the nasal cavities (128). NALT is considered to be a mucosal inductive site for humoral and cellular immune responses in the URT since it hosts B cell follicles surrounded by T cell areas (128, 129). In contrast to the LN, where naïve CD4^+^ T cells predominate over memory T cells, the NALT is surveyed primarily by memory CD4^+^ T cells, presumably resident type, suggesting that it is optimized to initiate memory recall responses, rather than initiate primary T cell responses (130). In contrast to memory CD4^+^ T cells in the NALT, CD8 T_RM_ cells tend to be distributed throughout the nasal turbinate and septum, although some antigen-specific CD8^+^ T_RM_ cells are also established in the NALT following recovery from a respiratory virus infection (131). In this regard, the distribution of T cells in the URT is similar to that in the skin and the FRT, where CD8^+^ T_RM_ cells are widely distributed in the epithelial tissues and CD4^+^ T_RM_ cells form clusters in the LP.

While the majority of CD8^+^ T_RM_ cells in the nasal tissues express CD103, a small fraction of the cells are CD103 negative (131). This differential expression of CD103 may reflect the localization of CD8^+^ T_RM_ cells within the epithelium and LP (132). Despite the high proportion of CD103^+^ cells in the URT, the differentiation of CD8^+^ T_RM_ cells in the nasal tissues does not appear to be dependent on local signaling through TGF-β and cognate antigen (43, 131, 133). This is in stark contrast to the LRT where both of these factors are absolutely required for the establishment of CD8^+^ T_RM_ cells (134, 135). Thus, the local instructions required for the differentiation of CD8^+^ T_RM_ cells in the nasal mucosa are distinct from those in the LRT. Furthermore, the number of CD8^+^ T_RM_ cells in the nasal tissues is relatively stable (there was no visible decline in number of these cells at least 3 months post-infection), whereas there is a significant decline in number of these cells in the LRT (lung) (131). This suggests that the nature of the anatomical niches that maintain CD8^+^ T_RM_ cells differ between URT and LRT. Given the structural similarity between nasal mucosa and other mucosal tissues and the fact that the nasal tissues retain γδ T cells in the epithelium (136), it is tempting to speculate that CD8^+^ T_RM_ cells in the nasal tissues may displace γδ T cells from their niches, potentially enabling their long-term survival.

The mucosal surfaces of the trachea and primary bronchus are basically similar to that of the nasal mucosa except for the presence of hyaline cartilage and a poorly developed venous plexus (the latter presumably helps avoid accidental suffocation caused by tracheal hemorrhage). Tracheal epithelial cells are a major target for several viral infections, such as seasonal influenza virus, and a recent study has demonstrated that large numbers of antigen-specific effector CD8^+^ T cells are recruited to the tracheal mucosa during the acute phase of the infection (137). By contrast, relatively few CD4^+^ T cells are recruited to the tracheal mucosa (as compared to the LRT) during the acute phase of infection. This suggests that there are distinct sets of homing signals in the mucosa of the trachea and LRT (137). Although establishment of CD8^+^ T_RM_ cells in the trachea was not determined in this study, CD8^+^ T cells were still detectable in the trachea following the resolution of an influenza virus infection (day 14), suggesting that some of these cells may reside in the tracheal epithelium as T_RM_.

The mucosa of the LRT is covered by pseudostratified ciliated epithelium (bronchiole) and columnar epithelium (terminal bronchiole to alveoli). A relatively thin interstitium underlies the epithelium and hosts both blood and lymphatic vessels. T cells in the LRT reside in at least two distinct compartments: the lung interstitium and the lung airways. T cells resident in the lung interstitium can be identified, and distinguished from circulating T cells, by intravenous labeling with an anti-T cell antibody (138). T cells in the lung airway are those that are collected by bronchoalevolar lavage taken *via* the trachea (139). Most of these cells are derived from the LRT (localized in the epithelial layer), although a few cells are also derived from the URT (trachea). CD8^+^ T cells exhibiting memory phenotypes can be detected in the LRT of naïve animals or animals that had previously been infected or vaccinated at sites distant from the lung (6, 43, 50, 140–143). It is believed that there is a basal level of influx that enables continual surveillance of the lung by antigen-experienced CD8^+^ T cells in the “lung-unconditioned” animals. For instance, some blood-borne cells are recruited to the airway under steady-state condition and CXCR3 expressed on antigen-experienced CD8^+^ T cells is known to be involved in this process (140). Once recruited to the lung airways, T cells do not return to the interstitium or the circulation unless there is an infection or an inflammatory condition (144).

Upon pulmonary infection, epithelial cells, lung-resident populations of immune cells in the interstitium and airway epithelium (such as macrophages, DC, and ILC) cooperatively promote acute inflammation (145). Although the full array of adhesion molecules and chemokine receptors that specifically guide T cells to the lung has not yet been determined, it is known that CXCR3 is important for the recruitment of effector CD8^+^ T cells to the epithelial layer of the interstitium as well as the airway (146). In addition, local inflammation-induced upregulation of CD69, and the activation of integrin α1β1 (very late antigen-1, CD49a) promotes transient localization and retention of CD8^+^ T cells in the lung interstitium (134, 147). As with the other mucosal tissues, local TGF-β signaling is required for the expression of CD103 on CD8^+^ T cells in the lung (135, 148), which then promotes localization of CD8^+^ T cells along the walls of large airways (149). IL-15 [produced primarily by CD11b^+^ macrophages in the interstitium during the early phases of a respiratory infection (150)] also facilitates the migration of effector CD8^+^ T cells to the lung (151). However, IL-15 is dispensable for the differentiation and maintenance of CD8^+^ T_RM_ cells in the lung (152).

**Figure 4:**
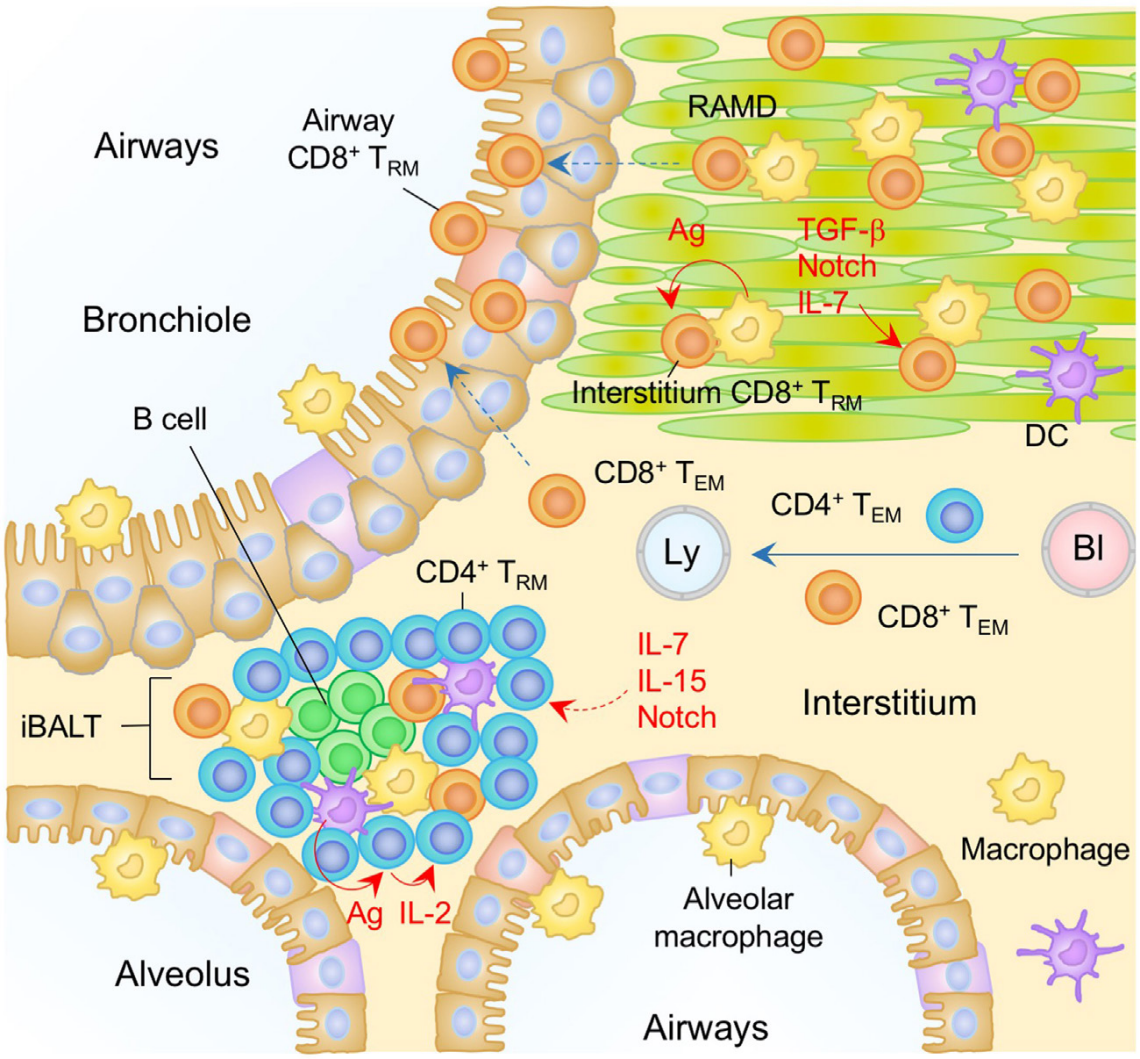
T_RM_ niches in the in the lung. A majority of CD8^+^ T_RM_ cells in the lung interstitium are maintained within the repair-associated memory depots (RAMD) that are temporarily created at the site of tissue injury, while CD8^+^ T_RM_ cells are found sparsely in the unaffected areas. A complex of niche factors, including signals *via* cognate antigen, TGF-β, Notch, and IL-7, are known to be involved in the formation of CD8^+^ T_RM_ cells in the lung interstitium. CD8^+^ T_RM_ cells are also present in the lung airways, the number of which is presumably maintained by continual recruitment of cells from the pool of CD8^+^ T_RM_ cells in the lung interstitium. CD4^+^ T_RM_ cells in the lung interstitium are maintained predominantly within the inducible bronchus-associated lymphoid tissues (iBALT). Late antigen recognition triggers autocrine IL-2 signaling, which supports the proliferation and survival of CD4^+^ T_RM_ cells. Homeostatic cytokines IL-7 and IL-15, and Notch signaling are also required for the maintenance of CD4^+^ T_RM_ cells in the iBALT. T_EM_ cells are passing through the normal interstitium. Orange and blue cells indicate CD8^+^ and CD4^+^ T_RM_ cells, respectively, unless otherwise stated. Red lines indicate the representative niche factors that influence the maintenance of T_RM_ cells. A blue line indicates the migratory route. Dashed lines indicate potential impact of niche factors (red) or migratory routes (blue). Abbreviations: Ly, lymph vessel; Bl, blood vessel; Ag, antigen; T_RM_, tissue-resident memory T cells; T_EM_, effector memory T cells.

Following the resolution of infection, substantial numbers of memory CD8^+^ T cells are maintained in both the lung interstitium and the airways for several months (153). We have recently shown that memory CD8^+^ T cells in both of these sites comprise a mixture of two distinct memory T cell populations: a major, stable population of T_RM_ cells, and a minor, dynamic population of T_EM_ cells that is continuously replenished by new cells from the circulation (134) (Figure 4). We also identified specific anatomical niches for CD8^+^ T_RM_ cells around the bronchiole, which are temporarily created at sites of regeneration following tissue injury (134). We termed these sites repair-associated memory depots (RAMD). As with the epithelial layers in other mucosal surfaces, CD8^+^ T_RM_ cells in the RAMD do not form clusters or lymphoid-like structures, but instead accumulate to relatively high densities in specific niches. By contrast, CD8^+^ T_EM_ cells are widely, but sparsely, distributed throughout the unaffected lung interstitium. This rigid compartmentalization of memory CD8^+^ T cell populations in the lung suggests that the two populations are maintained by separate signals. It is also important to note that residual antigen-driven reactivation in the mediastinal LN plays a role in driving the continual recruitment of CD8^+^ T_EM_ cells to the lung for several months after infection (154–157). Local instructive signals induced by pulmonary infection, such as IL-33 and TNF, presumably also contribute to the transient retention of circulating CD8^+^ T_EM_ cells in the lung interstitium (157). A more detailed analysis of the factors and mechanisms that regulate the continual recruitment of memory CD8^+^ T cells to the lung has been presented in our previous review (5).

Interestingly, in our parabiosis experiments we also detected minimal, if any, conversion of CD8^+^ T_EM_ cells into CD8^+^ T_RM_ cells in the lung for several months post-infection, a time period when T_RM_ cells still comprise a large proportion of memory CD8^+^ T cell pool in the lung (134). These studies further demonstrated that CD8^+^ T cells recruited to the lung interstitium after the peak of the cellular immune response (around day 10 post-infection) are excluded from the RAMD, and fail to form T_RM_ cells (134). These data clearly demonstrated that T_RM_ niches in the lung interstitium are occupied at the peak of tissue damage, but are no longer available for latecomer CD8^+^ T cells. In the skin and FRT sections, we noted that forced recruitment of CD8^+^ T cells to the epithelial tissues by antigen-independent inflammation or topical administration of chemokines results in the establishment of T_RM_ cells (prime and pull) (37, 127). Importantly, however, we and others have demonstrated that this prime and pull strategy does not work for the establishment of CD8^+^ T_RM_ cells in the lung, as CD8^+^ T cells recruited to the lung by antigen-independent inflammation in the lung completely disappear after the inflammation in the lung has resolved (134, 158). The failure of the prime and pull strategy in the lung is likely due to the structural difference between the lung and other mucosal/surface tissues. For instance, skin CD8^+^ T_RM_ cells can occupy DETC niches in the epidermis for their long-term survival, whereas normal lung mucosa does not exhibit such preformed niches. Administration of cognate antigen in combination with the prime and pull strategy results in the *de novo* creation of the RAMD, and significantly increases the numbers of antigen-specific, but not antigen-unrelated, CD8^+^ T_RM_ cells in the lung interstitium and airways (134). This indicates that local antigen plays at least two distinct roles: the creation of damage-associated niches by generating antigen-bearing target cells in the lung in the presence of antigen-specific CD8^+^ T cells in the circulation, and the antigen signaling necessary for the establishment, and/or survival, of T_RM_ (159). Following the establishment of T_RM_, Notch signaling may be a potential niche factor that regulates the maintenance of T_RM_ cells in the lung, as the lack of Notch signaling results in the loss of CD103^+^ CD8^+^ T_RM_ cells from the lung (160). Although cells that express Notch ligands are not yet identified in the RAMD, cell to cell contact seems important for sustaining T_RM_ cells in the lung. It is noteworthy that the size of the RAMD shrinks over time as tissue repair proceeds and tends to disappear several months post-infection (134). Such a transitional appearance of RAMD may account for the relatively shorter longevity of CD8^+^ T_RM_ cells in the lung (149). Recently, Zhou et al. have reported that the addition of local 4-1BB signaling during recall (4-1BB is expressed mainly on memory but not naïve T cells) improves the generation of long-lived CD8^+^ T_RM_ cells expressing IL-7 receptor (IL-7R)α (161), suggesting that IL-7 plays a key role in the maintenance of CD8^+^ T_RM_ cells in the lung. It will be interesting to determine whether these cells can survive outside the RAMD.

In contrast to the lung interstitium, the histological nature of putative CD8^+^ T_RM_ niches in the lung airways remains unclear. It has long been believed that the numbers of memory CD8^+^ T cells in the lung airways are maintained by the continual recruitment from the circulation. Resident cells at this site are cleared by phagocytic cells or removed through mucociliary clearance, resulting in a relatively short half-life (~2 weeks) (144). Surprisingly, our parabiosis experiments have demonstrated no evidence for the continual replacement of host memory CD8^+^ T cells in the lung airways by CD8^+^ T_EM_ cells derived from the partner. Since it is unlikely that memory CD8^+^ T cells can persist for long within the harsh airway environment, we assume that cells in the airways are continually replenished by CD8^+^ T_RM_ cells from the RAMD (interstitium) but not by CD8^+^ T_EM_ cells from the circulation. Thus, the major source of CD8^+^ T cells in the lung airways may be RAMD located underneath the bronchoalveolar walls (Figure 4).

In contrast to CD8^+^ T_RM_ cells, most CD4^+^ T_RM_ cells in the lung are found in B cell follicles and are surrounded by T cell areas (134, 162–164) (Figure 4). Such lymphoid-like structures have been termed inducible bronchus-associated lymphoid tissues (iBALT) and are the primary niches for the maintenance of lung CD4^+^ T_RM_ cells. The factors regulating the development of iBALT are reviewed elsewhere (165). Several other physiological niches for the generation and maintenance of lung CD4^+^ T_RM_ cells have also been reported. As with the CD8^+^ T_RM_ cells, local antigen also plays a role (163), as late antigen recognition at day 5–8 post-infection, which has been termed a “memory check point,” is necessary for the formation of memory CD4^+^ T cells in the lung and spleen (166). Antigen reactivation of the cells triggers autocrine IL-2 signaling, which prolongs the survival of CD4^+^ T_RM_ cells by upregulating the IL-7Rα (166–168) and sustains the homeostasis of lung CD4^+^ T_RM_ cells (162, 164). Interestingly, IL-15 signaling, as opposed to IL-2 signaling, can generate a separate but similar cohort of highly functional and protective CD4^+^ T_RM_ cells in the lung (169). As with the CD8^+^ T_RM_ cells, increased transcription levels of Notch signaling-associated molecules are observed in lung CD4^+^ T_RM_ cells, suggesting the involvement of Notch signaling for the maintenance of lung CD4^+^ T_RM_ cells (170).

#### Salivary Gland (SG)

The SGs are exocrine epithelial tissues that secrete saliva into the oral cavity. Humans and rodents have at least three pairs of major SGs (parotid, sublingual, and submandibular) and each gland has secretory units composed of an acinus, myoepithelial cells, and a duct (171). SGs also function as an effector site for IgA-mediated humoral immune responses that protect oral surfaces (172, 173).

**Figure 5:**
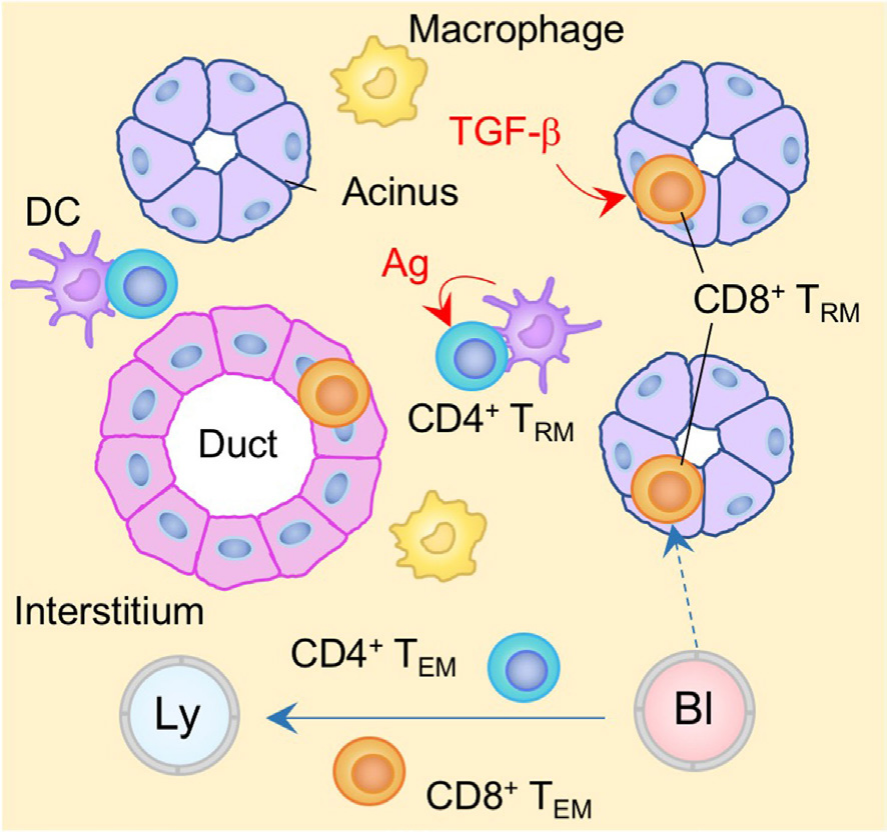
T_RM_ niches in the in the salivary gland (SG). CD8^+^ T_RM_ cells are localized predominantly within the epithelium of the acini and duct. Local TGF-β signaling is required for the formation of CD8^+^ T_RM_ cells in the SG. By contrast, CD4^+^ T_RM_ cells are localized predominantly within the stroma of the SG. In contrast to CD8^+^ T_RM_ cells, cognate antigen plays a key role in the formation of CD4^+^ T_RM_ cells. T_EM_ cells are passing through the normal interstitium. Red lines indicate the representative niche factors that influence the maintenance of T_RM_ cells. Orange and blue cells indicate CD8^+^ and CD4^+^ T_RM_ cells, respectively, unless otherwise stated. A blue line indicates the migratory route. A dashed line indicates potential migratory route. Abbreviations: Ly, lymph vessel; Bl, blood vessel; Ag, antigen; T_RM_, tissue-resident memory T cells; T_EM_, effector memory T cells.

It is well known that the SGs can be a target of a variety of bacterial as well as viral infections, such as mumps and cytomegalovirus (CMV). In the case of CMV, the virus is able to establish latent infection in the SGs and is able to evade CD8^+^ T cell immunity by downregulating MHC class I molecules (174). Virus-specific CD4^+^ T cells can control viral production, but are not able to eliminate latently infected cells (175, 176) such that persistent virus is selectively sequestered in the vacuoles of glandular acinar epithelial cells (177, 178). In latently infected individuals, resident populations of antigen-specific CD8^+^ and CD4^+^ T_RM_ cells are established in the SGs (179, 180) (Figure 5). However, their phenotypes, localization, and the local cues regulating their differentiation into T_RM_, differ significantly (181). CD4^+^ T_RM_ cells are located predominantly in the stroma of the SGs and their establishment depends on local antigen (179), presumably due to the upregulation of CD69 that antagonizes S1P_1_-medaited tissue egress (181). By contrast, CD8^+^ T_RM_ cells express CD103, and localize predominantly within the epithelium of the acini and ducts (179, 180) (Figure 5). Local TGF-β signaling in the SGs is required for upregulation of CD103 on CD8^+^ T_RM_ cells and their localization into the epithelium (179, 180). Because CMV downregulates MHC class I molecules, particularly in infected acinar glandular epithelial cells in the SGs, local antigen does not appear to be required for the formation of CD8^+^ T_RM_ cells in the SGs (179). Indeed, virus-specific CD8^+^ T cells can be established in the SGs even in the absence of virus infection in this tissue (6, 182, 183). Furthermore, ongoing presentation of late antigens by non-hematopoietic cells in the LN or by virus-uninfected APC (*via* cross-presentation) during CMV infection results in substantial and sustained expansion of antigen-specific CD8^+^ T cells in the circulation, a process known as memory inflation (184–187). Some of these memory CD8^+^ T cells are also converted into T_RM_ cells in the SGs on a continual basis (180). Blockade of CXCR3, or the genetic deletion of either integrin α4β1 or E-cadherin on CD8^+^ T cells reduces the accumulation of CD8^+^ T_RM_ cells in the SGs (182, 183, 188), suggesting that these molecules promote the migration of circulating CD8^+^ T cells to the glandular epithelium. In contrast to the inability of the primary CD8^+^ T cell response to control the virus infection, CD8^+^ T_RM_ cells resident in the SGs can confer protection upon recall by eliminating CMV infected non-epithelial cells, where CMV fails to achieve complete downregulation of MHC class I molecule (179).

### Non-Barrier Tissues

#### Brain

Owing to the presence of the blood–brain barrier (BBB), the blood–cerebrospinal fluid (CSF) barrier (BCSFB), and the CSF–brain barrier, the central nervous system (CNS) is regarded as an immune privileged site with severely limited ingress of blood-borne T lymphocytes. Relatively few, if any, T cells are present in the healthy brain parenchyma under non-inflammatory conditions (189). Consequently, the aberrant accumulation of T cells in the brain parenchyma has generally been considered to be a pathogenic condition. However, it is now becoming clear that the few peripheral T cells present in the brain in the absence of inflammation play key a role in surveying the CNS and keeping the infectious pathogens in check (190), as the lack of these cells can result in opportunistic infections in the CNS (191).

The choroid plexus (CP) is recognized as a major gateway for peripheral T cell access to the CNS (192, 193). The CP is comprised of fenestrated blood capillaries lacking endothelial tight junctions (192). Thus, the barrier properties of the BCSFB at this site rely only on the monolayer of epithelial cells interconnected by tight junctions—a structure permissive for immune cell transit (192). Consequently, around 150,000–750,000 immune cells are present in the CSF of healthy individuals, and more than 90% of the T cells present are antigen-experienced (193). Recent studies have identified a lymphatic vessel network lining the dural sinuses that drain CSF and allow the transit of immune cells from the adjacent subarachnoid space and brain interstitial fluid to the cervical LN (194, 195). This implies that there is the continual trafficking of T_EM_ cells between CNS (e.g., meninges and FSC) and the circulation (196). Nevertheless, the brain parenchyma essentially lacks lymphatic vessels and is mostly devoid of T cells under steady-state conditions.

**Figure 6:**
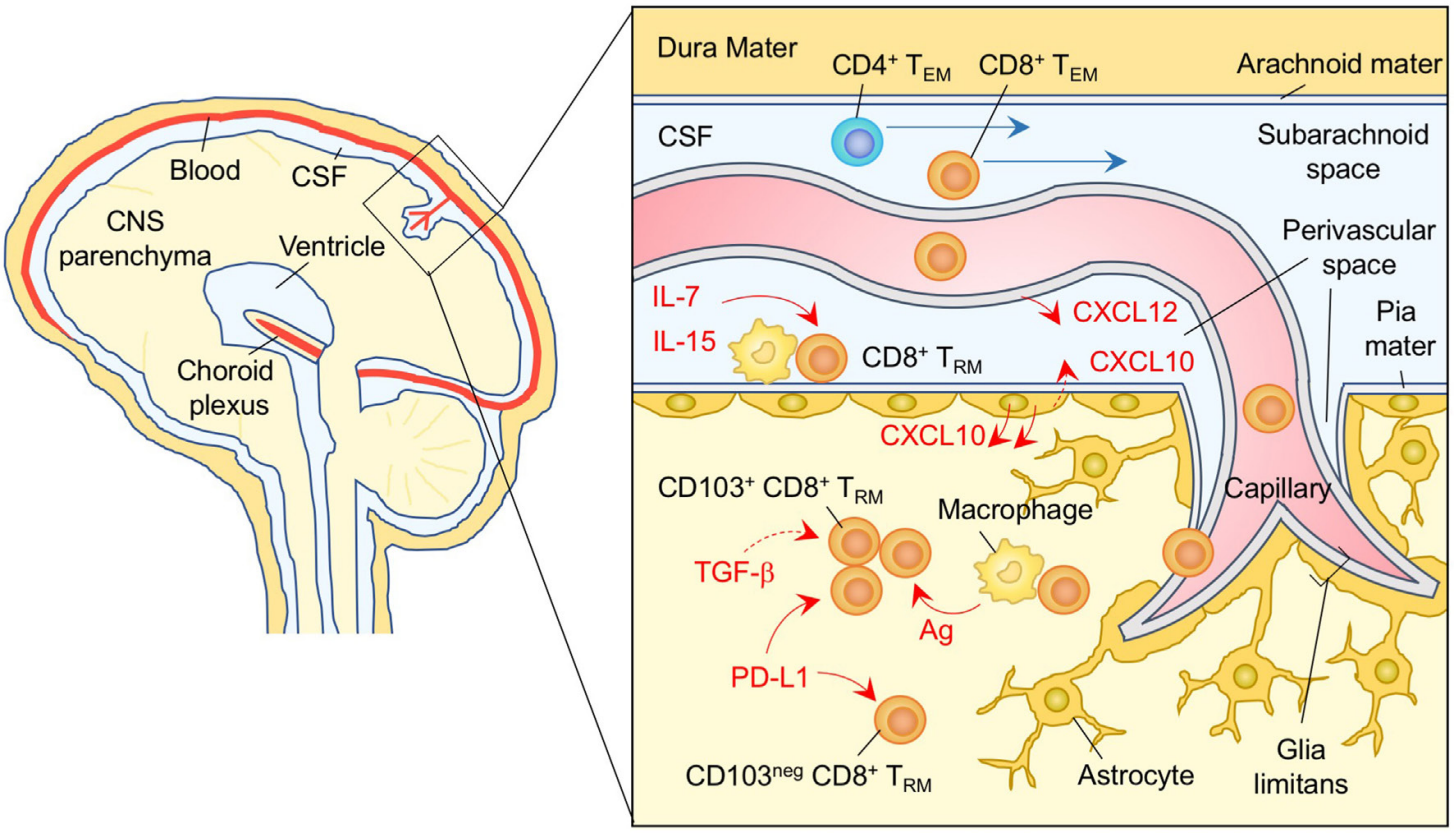
T_RM_ niches in the in the brain. CXCL12 is constitutively expressed on the basolateral surface of endothelial cells, and recruits CXCR4^+^ CD8^+^ T cells to the perivascular spaces. Subsequent migration of cells in the brain parenchyma is promoted by chemokines such as ligands for CXCR3 and CCR5 secreted by astrocytes. CD8^+^ T_RM_ cells are established in the parenchyma and perivascular spaces of the brain. Parenchymal CD8^+^ T_RM_ cells are divided by the expression of CD103. Although upregulation of CD103 depends largely on cognate antigen, TGF-β may be involved in this process. PD-1 is highly expressed on both CD103^+^ and CD103^−^ CD8^+^ T_RM_ cells in the brain parenchyma independent of either antigen or inflammatory signals. Signaling through PD-1 promotes the establishment of CD8^+^ T_RM_ cells in the brain parenchyma. IL-7 and IL-15 are available in the perivascular spaces, and sustain the homeostatic proliferation of CD8^+^ T_RM_ cells in this compartment. T_EM_ cells are passing through the cerebrospinal fluid (CSF). Orange and blue cells indicate CD8^+^ and CD4^+^ T_RM_ cells, respectively, unless otherwise stated. Red lines indicate the representative niche factors that influence the maintenance of T_RM_ cells. Blue lines indicate the migratory route. Dashed lines indicate potential impact of niche factors. Abbreviations: Ag, antigen; neg, negative; T_RM_, tissue-resident memory T cells; T_EM_, effector memory T cells.

Upon infection with neurotropic pathogens, antigen-specific T cells infiltrate the subarachnoid spaces of the meninges as well as the perivascular spaces of the parenchymal post-capillary venule, where specialized APC reside (197, 198) (Figure 6). T cells are then activated to proliferate and produce cytokines and chemokines in the infected meninges (199–201). This results in local inflammation, which subsequently disrupts vascular tight junctions and the glia limitans, allowing infiltration of T cells into the parenchyma (190, 198) (Figure 6). During this process, the balance of local chemokine production regulates the transmigration of circulating T cells into the brain parenchyma (202). In brief, CXCL12 is constitutively expressed on the basolateral surface of endothelial cell layer in the CNS and is also upregulated during inflammation, which promotes CXCR4^+^ T-cell recruitment to, and retention within, the perivascular space (203, 204). It is only after the local concentration of CXCL12 declines that effector T cells are able to migrate into the brain parenchyma in response to inflammatory chemokines, such as ligands for CXCR3 (205, 206) and CCR5 (207, 208). In the case of neuroinflammation associated with experimental autoimmune encephalomyelitis, the CXCL10–CXCR3 axis also functions to retain T cells within the perivascular space presumably due to differential inflammatory nature in the perivascular space (209).

After the clearance of a viral infection in the CNS, some of the antigen-specific CD8^+^ T cells that had been recruited to the brain parenchyma differentiate into T_RM_ cells and become resident in the site (133). The numbers of CD8^+^ T_RM_ cells that establish residency depends on the pathogen and is presumably linked to the tropism and pathogenesis of each virus (210). For example, following intranasal infection with vesicular stomatitis virus, which infects nerve endings, CD8^+^ T_RM_ cells form clusters at the site of infection, and are widely distributed throughout the brain parenchyma (133). By contrast, intracerebral infection with LCMV, which infects non-neuronal cells in the brain (i.e., glial cells), CD8^+^ T_RM_ cell populations are primarily established at brain surface structures, such as meninges and CP (around the ventricles or at anatomical borders between different brain regions) (211). In both cases, these CD8^+^ T_RM_ cell populations are not pathogenic, but confer protection against reinfection even in the absence of circulating memory CD8^+^ T cells (211).

Regardless of their location and the nature of the infecting pathogens, brain CD8^+^ T_RM_ cells can be divided into at least two populations based on their expression of CD103 (133, 211–214). It has been proposed that the initial upregulation of CD103 is largely dependent on the local reactivation of CD8^+^ T cells with cognate antigen in the brain (it remains elevated following antigen clearance) (133). However, it is clear that Treg-derived TGF-β (215, 216), inflammation, and other undefined local factors (213), can also upregulate CD103 on CD8^+^ T_RM_ cells in the brain in an antigen-independent manner. These different types of instructive signals may account for the distinct gene expression profiles between CD103^+^ and CD103^−^ CD8^+^ T_RM_ cells (212, 214) and the superior effector functions for the former (213, 214). It is noteworthy that retroviral knockdown of CD103 impairs the accumulation of CD8^+^ T_RM_ cells in the brain, indicating the importance of CD103 for the recruitment and/or retention of CD8^+^ T cells early after infection, probably during transmigration through the BBB. Once recruited to the brain parenchyma, however, CD103 expression has no impact on the localization of CD8^+^ T_RM_ cells (211), which may be attributed to the lack of E-cadherin expression in the adult brain (217). Taken together, it is possible that CD103 expressed on brain CD8^+^ T_RM_ cells may reflect the prior acquisition of local education but is not functional as an adhesion molecule.

Programmed cell death protein 1 (PD-1) and CD69 are both expressed on CD8^+^ T_RM_ cells in the brain (including both CD103^+^ and CD103^−^ T_RM_) (213). Although the expression of both molecules on CD8^+^ T_RM_ in non-CNS sites is generally dependent on repetitive antigen engagement (218), it has been demonstrated that both antigen and inflammation are dispensable for the sustained expression of PD-1 as well as CD69, and programmed cell death ligand 1 (PD-L1) in the brain (213). Furthermore, these cells remain functionally competent under these conditions (213). Interestingly, PD-1 expression on brain CD8^+^ T_RM_ cells is found to be programmed, as environmental factors in the brain induce extensive demethylation of the *Pdcd1* promoter (which controls PD-1 expression) (213). In addition, genetic deletion of either PD-1 or PD-L1 diminishes the establishment of brain CD8^+^ T_RM_ cells (219, 220). These findings suggest that signaling through PD-1 is a part of the T_RM_ differentiation program and may be attributed to the PD-1 signaling-induced upregulation of CPT1a, an enzyme necessary for fatty acid β-oxidation that promotes memory differentiation (221, 222). Since upregulation of PD-L1 expression is evident on parenchymal cells (e.g., microglia, astrocytes, and oligodendrocytes) following different types of viral infections in the CNS (223–227), it is reasonable to speculate that PD-1 expression by brain CD8^+^ T_RM_ cells maintains a tolerable balance between immunopathology and immune control of the virus in the CNS (190).

Reports of Ki-67 expression on brain CD8^+^ T_RM_ cells following resolution of virus infection suggests that these cells are maintained by homeostatic proliferation (211). CD8^+^ T_RM_ cells located at the brain surface structures more frequently express Ki-67 and phosphorylated Stat5 than those in the brain parenchyma, suggesting that their anatomical location allows them access to the homeostatic cytokines, IL-7 and IL-15 (211). Furthermore, CD8^+^ T_RM_ cells in the brain parenchyma are less responsive to homeostatic cytokines (212). Interestingly, CD8^+^ T_RM_ cells in the brain parenchyma, especially the CD103^+^ population, are not able to survive outside their tissue niche. The irreversible nature of tissue adaptation by CD8^+^ T_RM_ cells in the brain parenchyma is very different to the situation in the lung airway where CD8^+^ T_RM_ cells retain the plasticity to adapt to different environmental niches for their survival (133, 228).

#### Liver

The liver is a frontline immune tissue in which antigen-rich blood from the gastrointestinal tract enters *via* the portal vein and is passed through a network of sinusoids (the capillary bed of the liver). Antigens are effectively trapped by sinusoidal resident APC, such as Kupffer cells, liver sinusoidal endothelial cells (LSEC), and DC (229), and the relatively slow sinusoidal blood flow promotes effective interaction of circulating immune cells with these APC (230). Fenestrated sinusoidal endothelium also enables the direct surveillance of hepatocytes by circulating T cells (231).

**Figure 7:**
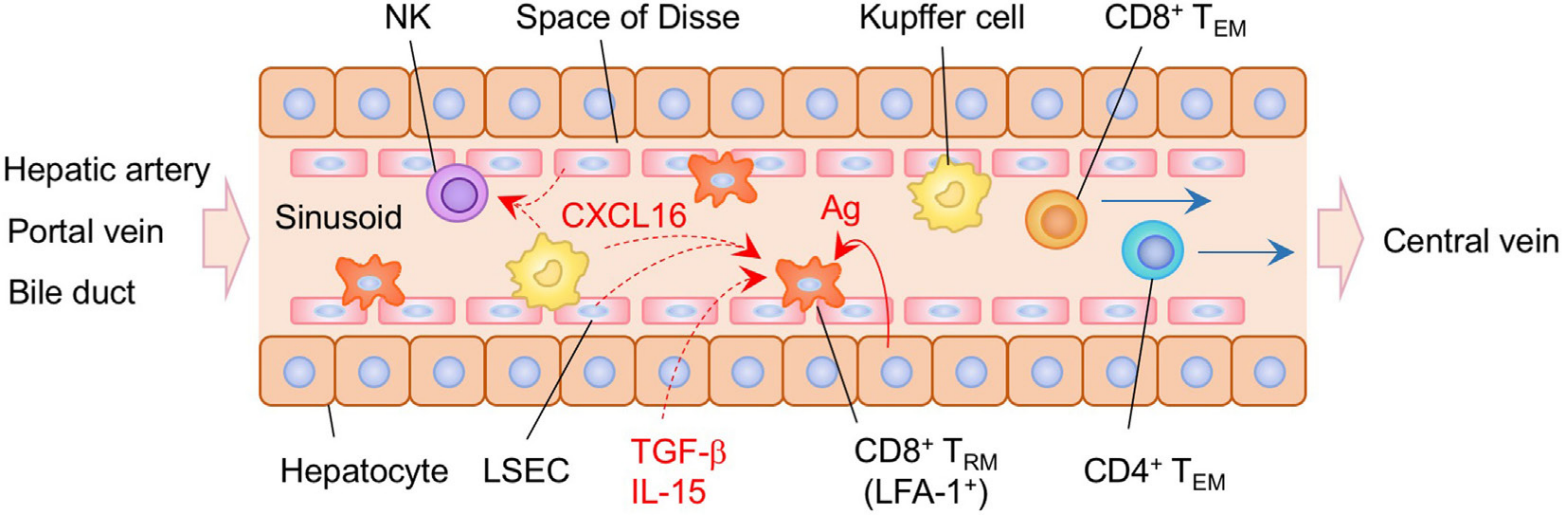
T_RM_ niches in the in the liver. CD8^+^ T_RM_ cells are localized within the sinusoid of the liver. These cells exhibit an amoeboid shape and crawling along the sinusoid dependent on the expression of lymphocyte function-associated antigen 1 (LFA-1). Antigen expressed on the hepatocytes play a key role in the establishment of CD8^+^ T_RM_ cells in the liver sinusoid. Kupffer cells and NK cells are also resident populations in the sinusoid. CXCL16 secreted by Kupffer cells and liver sinusoidal endothelial cells (LSEC) may promote the retention of T_RM_ cells and NK cells within the sinusoid. T_EM_ cells are passing through the sinusoid without crawling. Orange and blue cells indicate CD8^+^ and CD4^+^ T_RM_ cells, respectively, unless otherwise stated. A red line indicates the representative niche factor that influences the maintenance of T_RM_ cells. Blue lines indicate the migratory route. Dashed lines indicate potential impact of niche factors. Abbreviations: Ag, antigen; T_RM_, tissue-resident memory T cells; T_EM_, effector memory T cells.

Recent studies have demonstrated that liver-resident memory CD8^+^ T cells are established in the sinusoid following systemic infection or vaccination (232) (Figure 7). Liver CD8^+^ T_RM_ cells in mice are mostly CD69^+^, CXCR3^+^, and CXCR6^+^, but lack the expression of CD103, presumably reflecting the lack of tight junctions in the sinusoidal endothelium. The situation in humans is slightly different since a subset of CD8^+^ T_RM_ in the human liver are CD103^+^ in both healthy and hepatitis B virus-infected individuals. In this case, the sequential exposure of the cells to IL-15 and TGF-β induces the development of liver-adapted CD103^+^ CD8^+^ T_RM_ cells (233). Interestingly, mouse liver CD8^+^ T_RM_ cells exhibit an amoeboid shape and migrate with a crawling action along the sinusoids, whereas circulating CD8^+^ T_EM_ cells exhibit a round shape and flow rapidly in the sinusoid (232). Lymphocyte function-associated antigen 1 (LFA-1) has been found to be crucial for the patrolling behavior of liver CD8^+^ T_RM_ cells in the sinusoid (234). It is also known that Kupffer cells, macrophages, and LSEC in the sinusoid constitutively express CXCL16, a CXCR6 ligand (235–237), which attracts NK cells, another resident cell population in the sinusoid (238). This suggests that liver-resident CD8^+^ T cells and NK cells share this chemokine niche (239), although competition between these populations for this niche has not been reported. Local antigen presentation is clearly important for the prolonged retention and establishment of CD8^+^ T_RM_ cells in the sinusoid, as targeting antigen presentation to the hepatocytes in the presence of antigen-specific CD8^+^ T cells in the circulation leads to the massive accumulation of CD8^+^ T_RM_ cells in the sinusoid, a strategy termed as “prime and trap” (232). Since local antigen presentation in the liver can trigger the formation of tertiary immune structures known as intrahepatic myeloid cell aggregates for T cell population expansion (iMATE) (240), it is tempting to speculate that such follicle-like structures provide special T cell niches in the liver, especially for CD4^+^ T_RM_ cells.

#### Kidneys

The kidney is a highly vascularized tissue that is crucial for filtering the blood and removing toxins from the body. Lymphocytes are relatively rare in healthy kidneys, although small numbers of resident immune cells such as DC, macrophages, and T cells can be found in the interstitium under steady-state condition (241, 242). CD8^+^ T_RM_ cells can persist in extravascular renal compartments following direct (243) or regional infections with pathogens (6, 83, 99, 244), although their precise distribution is not clear (244). While the majority of renal CD8^+^ T_RM_ cells express CD69, even in the absence of antigen, only small fraction of cells express CD103 (83, 243, 244). The tissue-derived factors that influence the formation of renal CD8^+^ T_RM_ cells are poorly defined. However, it has been reported that a lack of TGF-β signaling leads to reduction in the formation of CD8^+^ T_RM_ cells in the kidney (244). This has been attributed to the role of TGF-β signaling in promoting trans-endothelial migration of effector CD8^+^ T cells by upregulating ligands for E- and P-selectin, including an activated form of CD43, and CXCR3 (244). IL-15 is also known to be essential for the upregulation of CD43 (245), which may explain the defective establishment of renal CD8^+^ T_RM_ cells in the absence of IL-15 (99).

#### White Adipose Tissue (WAT)

While T_RM_ generally function locally to guard the vulnerable sites from reinfection, an interesting exception is the establishment of antigen-specific CD8^+^ T_RM_ cells in the WAT (246). These cells exhibit a high turnover rate and active metabolism and can augment recall responses generated by non-lipid compartments, suggesting that the WAT functions as a reservoir of T_RM_ cells by improving their functional capacities and longevity. Notably, WAT T_RM_ cells also remodel the physiological function of the WAT, as reactivation of adipose T_RM_ cells lead to a sharp decrease in lipid synthesis. This elevates the antimicrobial responses within the adipose tissues, resulting in synergic immunological crosstalk between the tissue and the T_RM_ cells. Thus, it is of interesting to speculate that, beyond the role as the local sentinel, long-term maintenance of T_RM_ cells may influence the homeostasis and function of each tissue, leading to both beneficial and detrimental consequences.

#### Tumor

It has been reported that CD8^+^ T cells with a T_RM_ phenotypes (CD103^+^ and CD49a^+^) are present in solid tumors (247, 248). Large-scale transcriptome analysis has revealed that CD8^+^ tumor infiltrating lymphocytes (TIL) exhibit characteristics of T_RM_ cells and it has been observed that CD103^+^ CD8^+^ T_RM_ cells from neighboring peripheral tissues can infiltrate into solid tumors (249, 250). Runx3 expression appears to promote the infiltration of CD8^+^ T_RM_ cells into tumors as *Runx3*-deficient CD8^+^ T cells failed to accumulate in tumors (90). As with other tissues, local microenvironmental cues promote the acquisition of T_RM_ phenotypes of CD8^+^ that infiltrate tumor tissues (251). It is important to note, however, that CD8^+^ TIL with T_RM_ characteristics (termed as CD8^+^ T_RM_ TIL hereafter) are no longer true “resting” T_RM_ cells as they are located in an effector site where cognate antigen is abundant and typically express checkpoint molecules to regulate their activity (249). This checkpoint molecule expression may be transient, or below suppressive levels, since CD8^+^ T_RM_ TIL in tumors exhibit superior anti-tumor activities and a positive prognosis has been correlated with the quality and quantity of these cells (248–250, 252–256). It has also been found that CD103^+^ CD8^+^ T_RM_ TIL with the strongest CTL activity are located in the border area of the tumor. This contrasts with CD103 negative CD8^+^ T_RM_ TIL that infiltrate the stroma of the tumor (a potentially highly immune suppressive environment), and mediate weak CTL activity (257). CD103-mediated efficient interaction of CD8^+^ T_RM_ TIL with tumor cells of epithelial origin also promotes prolonged survival and enhanced CTL activity (251, 254, 258, 259). Based on these findings, the generation of CD8^+^ T_RM_ cells in neighboring tissues to the tumor is a promising strategy to confer protection against tumor growth (250, 260–263). However, this protection is limited to primary tumors, and not metastases, since CD8^+^ T_RM_ cells are segregated from the circulation (250).

## Lymphoid Organs

### Secondary Lymphoid Organs

#### LNs, Spleen

The SLOs have generally been considered a transit site for T_CM_ and T_EM_ cells. In the case of the LN, these cells are transiting from the high endothelial venules and afferent lymphatics, respectively, into the circulation. However, recent studies have demonstrated that there are also small numbers of memory CD4^+^ and CD8^+^ T cells that are resident in the LN, spleen, PP, and tonsils without recirculation (264–268). The long-term residency of T_RM_ cells within the SLO has been demonstrated by parabiosis or photoconversion-based cell labeling studies (264, 265, 267, 268). Unlike circulating memory T cells, T_RM_ cells in the SLO share phenotypic characteristics and gene expression profiles with those in the NLT (110), including stable downregulation of S1P_1_, a key molecule for regulating T-cell egress from the LN (55). Indeed, most T_RM_ cells in the SLO express CD69, which promotes the downregulation of S1P_1_ (110, 264, 266, 268). Since surface expression of CD69 is generally transient, however, it is likely that repetitive antigen stimulation is required for the maintenance of CD69 expression and the retention of T_RM_ cells in the SLO (110). In this regard, there is considerable evidence that residual antigen persists in the draining LN for several months after vaccination or the resolution of an acute infection and presumably facilitates the accumulation of memory T cells (154–156, 269–272). In addition, a recent study by Beura et al. have demonstrated that some CD8^+^ T_RM_ cells in the LN are derived from cells that exit the NLT (273), thereby enhancing the accumulation of antigen-specific CD8^+^ T_RM_ cells in the draining LN.

**Figure 8:**
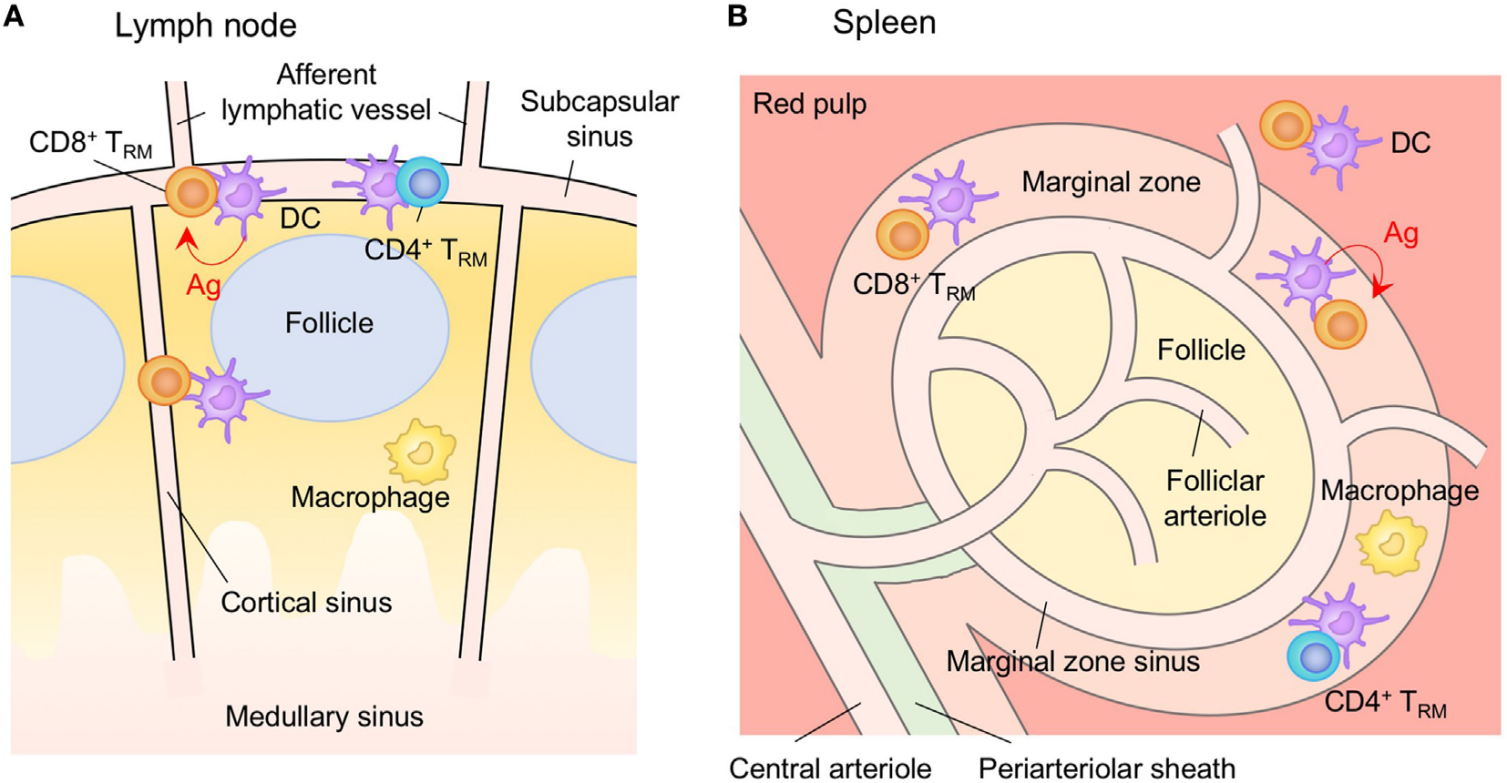
T_RM_ niches in the in the secondary lymphoid organs (SLOs). Both CD4^+^ and CD8^+^ T_RM_ cells in the SLO are found at the common antigen entry sites, such as the subcapsular sinus in the lymph nodes **(A)**, and the marginal zone and red pulp in the spleen **(B)**. Retention of cells in these compartment is largely dependent on the expression of CD69 in response to antigen, although retention induced by CD69-independent mechanisms is also suspected. Orange cells indicate CD8^+^ T_RM_ cells unless otherwise stated. Red lines indicate the representative niche factors that influence the maintenance of T_RM_ cells. Abbreviations: Ag, antigen; T_RM_, tissue-resident memory T cells.

The distribution of T_RM_ cells in the SLO depends on an antigen niche, as T_RM_ cells are preferentially localized at the common antigen entry sites: the marginal zone and red pulp of the spleen and the subcapsular sinuses of the LN (264) (Figure 8). Although the maintenance of murine T_RM_ cells in the SLO is relatively independent of IL-15, signaling *via* IL-15 and TGF-β are known to transcriptionally downregulate S1P_1_ in human T cells. Indeed, T_RM_ cells in the tonsils are localized specifically near the epithelial barrier where IL-15 is constitutively expressed (266). This is indicative of cytokine niche-dependent compartmentalization of T_RM_ cells within the SLO. Since T_CM_ cells in the SLO are central to pathogen clearance by generating massively increased numbers of secondary effector T cells during a recall response, it will be important to determine the functional contribution of T_RM_ cells in the SLOs during the recall responses. It is possible that T_RM_ cells in the SLO do not actively contribute to the recall response to avoid unnecessary competition with T_CM_ cells, but are strategically positioned to protect the SLO from direct infection with pathogens.

### Primary Lymphoid Organs

#### Thymus

**Figure 9:**
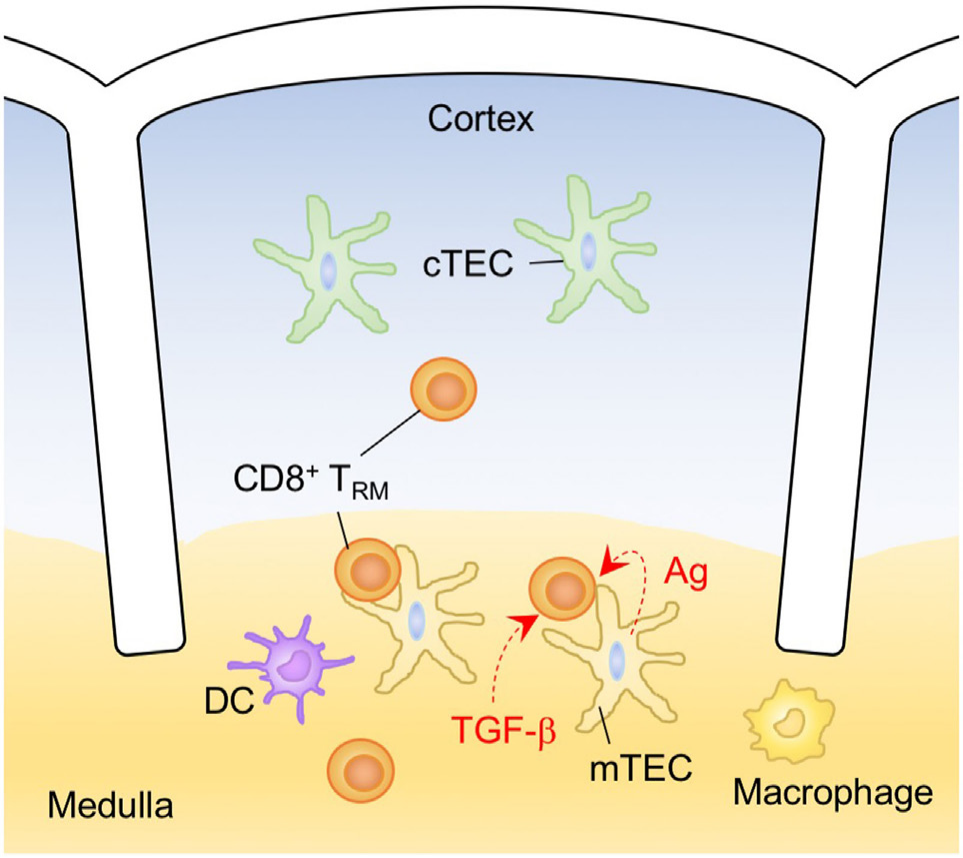
T_RM_ niches in the in the thymus. CD8^+^ T_RM_ cells are localized predominantly within the thymic medulla, although some cells are also found in the cortex. A majority of CD8^+^ T_RM_ cells in the thymus express CD103 and CD69. TGF-β is rich in the medulla, and presumably influences the CD8^+^ T cell expression of CD103. Since persistent presentation of foreign antigen in the thymus is uncommon, self-antigen may drive the expression of CD69 on thymic CD8^+^ T_RM_ cells. Orange cells indicate CD8^+^ T_RM_ cells unless otherwise stated. Dashed lines indicate potential impact of niche factors. Abbreviations: Ag, antigen; cTEC, cortical thymic epithelial cell; mTEC, medullary thymic epithelial cell; T_RM_, tissue-resident memory T cells.

Antigen-specific CD8^+^ T_RM_ cells have also been found to persist in the thymus, a primary lymphoid organ (274). Thymic CD8^+^ T_RM_ cells are established following infection with either thymus-tropic or non-tropic pathogens, with considerably higher numbers in the former. As with T_RM_ cells in the peripheral tissues, thymic CD8^+^ T_RM_ cells exhibit a canonical T_RM_ phenotype (CD69^+^ CD103^+^). These cells localize predominantly in the medulla although a few cells lodge in the cortex (Figure 9). At least three mechanisms potentially explain the medullary localization of thymic CD8^+^ T_RM_ cells. First, active TGF-β, which support the generation of thymic Treg cells and potentially upregulates T cell expression of CD103, is predominantly localized in the thymic medulla (275). Second, E-cadherin is highly expressed in all thymic epithelial cells (TEC) of both the cortex and medulla (276) and promotes the interaction of TEC with CD103^+^ thymocytes (277). Third, mature thymocytes express CD69 which induces the downregulation of S1P_1_ on CD8^+^ T_RM_ and blocks the departure of the cells *via* the medulla or cortico-medullary junction (278). The factors that induce the upregulation of CD69 on thymic CD8^+^ T_RM_ cells have not been determined (274). Since the immune activation process strongly inhibits the migration of peripheral DC populations to the thymus to avoid unfavorable induction of acquired tolerance to the invading pathogens (279, 280), it is reasonable to think that thymic CD8^+^ T_RM_ cells mainly function to protect the thymus, rather than contribute to the recall responses against systemic infections.

#### Bone Marrow (BM)

The BM is another primary lymphoid organ that facilitates the long-term maintenance of memory T cells by providing at least two distinct niches: a quiescence niche, that harbors a majority of quiescent memory T cells, and a self-renewal niche where memory T cells undergo homeostatic proliferation (281). Indeed, large numbers of memory CD8^+^ and CD4^+^ T cells accumulate in the BM (282, 283) and most of them express high levels of CD69, a hallmark of T_RM_ cells (164, 284, 285). TGF-β, secreted mainly by megakaryocytes in the BM, regulates the quiescence of memory T cells (286) and CXCL12 produced by reticular stromal cells promotes their co-localization with CXCR4^+^ memory T cells (287). The reticular stromal cells, as well as myeloid cells, in the BM also provide niche factors for self-renewal such as IL-7 and IL-15 (283, 288, 289).

Recently, Di Rosa and Gebhardt have speculated that memory CD8^+^ T cells in the BM are a circulating population that is transiting through the BM niches without establishing residence (290). This is largely based on the observation that memory CD8^+^ T cells derived from the host and partner equilibrate in the BM in parabiosis experiments (65). By contrast, the deposition of memory CD4^+^ T cells in the BM is relatively stable, as these cells persist in the BM for a long period even after most memory CD4^+^ T cells disappear from the spleen and LN (283). Interestingly, BM memory CD4^+^ T cells preferentially home back to the BM after adoptive transfer (283). A fraction of adoptively transferred splenic CD8^+^ T cells, particularly those with a memory phenotype, also home to the BM (282, 284, 291). These data suggest that circulating memory T cells have high levels of access to BM niches. High levels of access of memory T cells to the BM niches could also explain the low detection of T_RM_ cells in the parabiosis experiments. More analyses are required for precise characterization of T_RM_ cells in the BM.

## Concluding Remarks

The regulation, generation, and maintenance of T_RM_ cells depends on two primary cell-extrinsic factors: (i) local signals that enable microenvironmental adaptation of T cells in each tissue and (ii) the availability of tissue-specific anatomical niches. Non-immune cells as well as immune cell populations resident in each microenvironment provide these niche factors. Once established, T_RM_ cells function locally to guard the vulnerable sites from reinfection. Hence, a deep understanding the comprehensive picture of T_RM_ niches is required for the development of tissue-targeted vaccination strategies to effectively generate T_RM_ cells in each tissue. For example, “prime and pull” is a potential vaccination strategy for the skin and FRT, where T_RM_ cells can utilize niches that are originally occupied by other resident cells (37, 127). In sharp contrast, this strategy does not work for the lung due to the absence of preformed niches for T_RM_ cells to displace (134, 158). The creation of *de novo* niches in the lung by “prime and pull plus cognate antigen” partly resolves this problem (5, 134, 158). Antigen-niches also play a role in the establishment of T_RM_ cells in the vascularized tissues of the liver, a strategy referred to as “prime and trap” (232).

The description of T_RM_ niches in this review is based primarily on findings from mouse studies with occasional reference to work in humans. It is important to note, however, that the characteristics of T_RM_ cells in these species can vary. For example, the T_RM_ signature in humans is primarily defined by CD69^+^ expression (292), while CD69 expression is insufficient to infer tissue residence in mice (6, 273). Furthermore, a key transcription factor Hobit that instructs tissue residency is highly expressed by murine T_RM_ cells (62), while its expression is relatively low in human T_RM_ cells (292–294). These, and other, species differences in T_RM_ indicate that many more studies in humans will be necessary for the development of effective vaccines in the clinic.

In summary, the factors regulating the formation of T_RM_ cells in each tissue and each species are far more complex than originally thought, and numerous hurdles exist in generating and maintaining T_RM_ cells in each tissue in terms of the efficacy, safety, and longevity. There is still much to learn.

## Author Contributions

ST participated in the concept, wrote the manuscript, and developed the figures.

## Conflict of Interest Statement

The author declares that the research was conducted in the absence of any commercial or financial relationships that could be construed as a potential conflict of interest.
